# Intratumor genetic heterogeneity and clonal evolution to decode endometrial cancer progression

**DOI:** 10.1038/s41388-022-02221-0

**Published:** 2022-02-10

**Authors:** Alba Mota, Sara S. Oltra, Pier Selenica, Cristian P. Moiola, Carlos Casas-Arozamena, Carlos López-Gil, Eva Diaz, Sonia Gatius, María Ruiz-Miro, Ana Calvo, Alejandro Rojo-Sebastián, Pablo Hurtado, Roberto Piñeiro, Eva Colas, Antonio Gil-Moreno, Jorge S. Reis-Filho, Laura Muinelo-Romay, Miguel Abal, Xavier Matias-Guiu, Britta Weigelt, Gema Moreno-Bueno

**Affiliations:** 1 MD Anderson International Foundation, 28033 Madrid, Spain; 2 Biochemistry Department, Universidad Autónoma de Madrid (UAM), Instituto de Investigaciones Biomédicas ‘Alberto Sols’ (CSIC-UAM), IdiPaz, 28029 Madrid, Spain; 3 Centro de Investigación Biomédica en Red de Cáncer (CIBERONC), 28029 Madrid, Spain; 4 Department of Pathology, Memorial Sloan Kettering Cancer Center, New York, NY 10065 USA; 5 Biomedical Research Group in Gynecology, Vall Hebron Institute of Research, Universitat Autònoma de Barcelona, 08035 Barcelona, Spain; 6 Translational Medical Oncology Group (Oncomet), Health Research Institute of Santiago de Compostela (IDIS), University Hospital of Santiago de Compostela (SERGAS), Trav. Choupana s/n, 15706 Santiago de Compostela, Spain; 7 Department of Pathology, Hospital U Arnau de Vilanova, University of Lleida, IRBLLEIDA, Lleida, Spain; 8 Biobank, IRBLLEIDA, Lleida, Spain; 9 Department of Gynecology, Hospital U Arnau de Vilanova, IRBLLEIDA, Lleida, Spain; 10 MD Anderson Cancer Center, Madrid, Spain; 11 Roche-Chus Joint Unit, Translational Medical Oncology Group (Oncomet), Health Research Institute of Santiago de Compostela (IDIS), Travesía da Choupana s/n, 15706 Santiago de Compostela, Spain; 12 Gynaecological Department, Vall Hebron University Hospital, 08035 Barcelona, Spain; 13 Departments of Pathology, Hospital U. de Bellvitge, Universities of Lleida and Barcelona, IDIBELL Lleida and Barcelona, Spain

**Keywords:** Gynaecological cancer, Cancer genomics

## Abstract

Analyzing different tumor regions by next generation sequencing allows the assessment of intratumor genetic heterogeneity (ITGH), a phenomenon that has been studied widely in some tumor types but has been less well explored in endometrial carcinoma (EC). In this study, we sought to characterize the spatial and temporal heterogeneity of 9 different ECs using whole-exome sequencing, and by performing targeted sequencing validation of the 42 primary tumor regions and 30 metastatic samples analyzed. In addition, copy number alterations of serous carcinomas were assessed by comparative genomic hybridization arrays. From the somatic mutations, identified by whole-exome sequencing, 532 were validated by targeted sequencing. Based on these data, the phylogenetic tree reconstructed for each case allowed us to establish the tumors’ evolution and correlate this to tumor progression, prognosis, and the presence of recurrent disease. Moreover, we studied the genetic landscape of an ambiguous EC and the molecular profile obtained was used to guide the selection of a potential personalized therapy for this patient, which was subsequently validated by preclinical testing in patient-derived xenograft models. Overall, our study reveals the impact of analyzing different tumor regions to decipher the ITGH in ECs, which could help make the best treatment decision.

## Introduction

Endometrial cancer (EC) is the most common gynecological cancer in developed countries, and it accounts for nearly 5% of cancer cases and more than 2% of cancer-associated deaths in women worldwide [1]. EC has traditionally been classified into two main groups, type I and type II carcinomas, with different endocrine, clinical, pathological, and molecular features [2, 3]. More recently, The Cancer Genome Atlas (TCGA) defined a molecular classification of EC [4] based on somatic mutations, copy number alterations and microsatellite instability status. Four distinct EC molecular subtypes were established, associated with distinct outcomes, namely the POLE/ultramutated, microsatellite instable (MSI)/hypermutated, copy-number low (CN-low)/endometrioid and copy-number high (CN-high)/serous-like subtypes. Although this TCGA study improved the existing classification of ECs, there are still rare EC histologies that are challenging to classify. There is a ‘grey zone’ between grade 3 endometrioid and serous carcinomas, with some cases exhibiting overlapping morphological and molecular features [5, 6]. For example, high-grade ambiguous EC (AEC) has mixed, overlapping features or lack of any evidence of differentiation at both the microscopic and molecular levels [5–7], and it may not always fit into the molecular TCGA subtypes or the traditional dualistic histologic classification [5, 6]. These tumors have been previously described by other authors and account for less than 1% of all ECs [5–7]. They are often associated with aggressive behavior [7] and with the lack of response to conventional therapies [8]. The diagnosis of AEC is difficult to make in the absence of an intense molecular analysis, as performed in this study. AEC cases are occasionally placed in the spectrum of high-grade endometrioid carcinomas, although they are very aggressive, and do not fit perfectly in any TCGA subgroup. For these reasons, it is important to be aware of the existence of this unusual type of tumor.

Intratumor genetic heterogeneity (ITGH) has emerged based on the analyses of different areas of the primary tumor and metastatic lesions from the same patient using whole-exome sequencing (WES) and targeted massively parallel sequencing [9–12]. In this regard, we detected strong ITGH in primary ECs [13, 14], as previously described for other tumor types [15]. However, these studies did not address the spatial and temporal genetic heterogeneity among ECs, nor that between ECs and their distant metastases. Notably, ITGH analysis allows for the identification of sub-clonal variants present at low frequencies and non-uniform distribution across tumor regions. These variants could be important elements in tumor evolution and subsequently, in the patient’s response to treatment and for their prognosis [11, 16–18].

To get deeper insights into the behavior of ECs and to be able to adapt the clinical management of EC patients accordingly, it is imperative to decode the genomic evolution of these tumors. Thus, we sought here to define the spatial and, in some cases, temporal heterogeneity of ECs, their clonal composition, and the clonal shifts between primary and metastatic disease. Spatially distinct samples of primary tumors and metastatic lesions from five patients with metastatic endometrioid EC (EEC; three of which were MSI), three patients with metastatic serous EC (SECs), and one patient with high-grade AEC, were subjected to WES, targeted massively parallel sequencing, and a subset to array comparative genomic hybridization (aCGH) to decipher their genomic landscape. These data were employed to reconstruct the spatial and temporal, when was possible, evolution of the tumors within each patient. Several phylogenetic patterns were identified in these tumors, independent of their classic histological or molecular classification, including similar patterns in cases with ovarian metastasis or recurrent disease. Despite the abundant spatial ITGH within the primary ECs, the majority of anatomically distinct EC metastatic lesions from a given patient displayed genomic homogeneity. In addition, we defined the molecular evolution in an AEC tumor. Interestingly, our analyses on AEC identified new potential targetable biomarkers that were validated by treating a patient-derived xenograft (PDX) with specific therapies. Overall, our results could help to prompt new paths for the personalized treatment of EC patients and potentially for a rare subtype of these cancers like AEC.

## Results

### Clinicopathological characteristics of ECs included in this study

Nine metastatic EC patients with complete clinical follow-up information were included in this study: 5 endometrioid ECs (EEC3-7), 3 serous ECs (SEC1-3) and 1 AEC. Immunohistochemistry (IHC) analysis of the DNA mismatch repair (MMR) proteins and p53 (Supplementary Fig. 1A), combined with the *POLE* sequencing results (Table 1), were employed to define the ProMisE molecular subtypes [19, 20]. This analysis revealed that none of the nine ECs included in this study harbored a *POLE* hotspot mutation (data not shown), that 3/5 EECs lacked *MLH1* and *PMS2* expression and were classified as MSI molecular subtype, that 2/5 EECs were classified as CN-low, and that all 3 SECs showed aberrant p53 expression and were classified as CN-high (serous-like) (Table 1).

**Table 1:** Clinicopathological characteristics of the endometrial cancer patients included in the study.

Patient	Histology	Grade	FIGO	Myometrial infiltration	Lymph node invasion	POLE status	MSI status	TP53 IHC^a^	TCGA classification	Metastasis	Status^b^	RFS (years)	OS (years)
EEC-3	Endometrioid	2	IB	80%	No	WT	Negative	1+	CN-low	Iliac node	AWD	3.1	10.9
EEC-4	Endometrioid	2	IA	40%	No	WT	Positive-low	1+	CN-low	Peritoneum	DF	3.12	10.18
EEC-5	Endometrioid	3	IV	100%	Yes	WT	Positive-high	2+	MSI	Peritoneum	DOC	0.26	10.37
EEC-6	Endometrioid	3	IB	75%	No	WT	Positive-high	1+	MSI	Ovary	DOD	1.21	6.73
EEC-7	Endometrioid	3	IB	<50%	No	WT	Positive-high	1+	MSI	Diaphragm	AW	7.81	12.73
SEC-1	Serous	3	IIIB	95%	–	WT	Negative	0	Serous-like	Ovary	DOD	0.21	1.57
SEC-2	Serous	3	IIIA	80%	–	WT	Negative	2+	Serous-like	Ovary	AWD	0.49	1.51
SEC-3	Serous	3	IIIA	<50%	No	WT	Negative	2+	Serous-like	Ovary	DOD	1.4	2.32
AEC	Ambiguous	3	IIIC	95%	Yes (11/36)	WT	Negative	1+	Serous-like	Node	DOD	0.97	2.25

*PDX* patient-derived xenograft, *WES* whole-exome sequencing, *CNA* copy number aberration, *P* primary tumor, *M* metastasis.

^a^TP53 IHC: 2+ = p53 overexpression, indication of missense mutation; 1+ = wild-type pattern; 0 = absence of protein, indication of null mutation.

^b^*AW* alive and well, *AWD* alive with disease, *DF* disease free, *DOC* died of other cause, *DOD* died of disease.

All patients received the conventional treatment caboplatin-Taxol.

The AEC tumor displayed ambiguous histologic characteristics and when the ProMisE molecular surrogate was applied, the tumor was classified as CN-low or as a tumor with a non-specific molecular profile [20, 21]. Moreover, conventional histopathological and IHC assessment defined a high-grade carcinoma that did not fulfill the typical features of an endometrioid, serous, clear cell, or undifferentiated carcinoma (Supplementary Fig. 1A). After having been assessed by two blinded expert gynecological pathologists, the high confidence score (0.548) obtained in the TumorTracer study [22] confirmed the endometrial origin of this tumor by comparing the mutational profile of our AEC with that of more than 7600 tumors (Supplementary Fig. 1B). This tumor exhibited a solid pattern of growth with an absolute lack of any type of differentiation (endometrioid, serous, clear cell). In addition, although AEC could misclassify with undifferentiated carcinoma, did not show any of the molecular and immunohistochemical features of these tumors [23]. These data provide further evidence that the tumor has an endometrial origin as opposed to metastasis of an extrauterine malignancy. Additionally, HPV testing was also negative, ruling out an origin in the uterine cervix (Supplementary Fig. 1C). In light of the complexity of the AEC tumor and to corroborate its phenotype, an additional IHC study was performed with a panel of biomarkers previously used to differentiate between high-grade endometrioid and serous carcinoma (see Fig. 6A, for more details) [24]. This analysis was inconclusive for all the tumor areas tested and thus, the tumor was classified as a non-specific AEC tumor. Indeed, based on the gross, microscopic and IHC data, this tumor was confirmed as a high-grade EC with ambiguous features, FIGO stage IIIC2.

Although small sample size, comparison based on TCGA classification (including CN-low *n* = 2, MSI *n* = 3, serous *n* = 3 and AEC *n* = 1) and histological type (Endometrioid *n* = 5, Serous *n* = 3 and AEC *n* = 1) revealed significantly different overall survival rates (Supplementary Fig. 1D, *p* = 0.025 and *p* = 0.01, respectively). Interestingly, the case of AEC and serous tumors were associated with the poorest survival. Even though, these results should be confirmed in a larger cohort, our analysis supported previous studies that showed an association of endometrioid cases with low-intermediate-risk and serous-like groups with the worst outcomes [25].

## The mutational repertoire identified by WES reveals a profile consistent with EECs and SECs

**Fig. 1:**
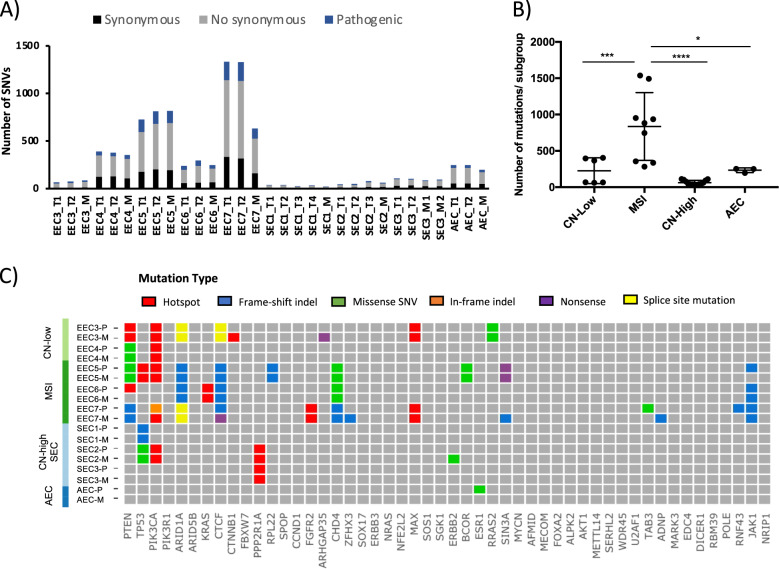
Immunohistochemical and genomic representations of endometrial carcinomas. **A** Graphical representation of the somatic variants (SNVs) detected by samples analysis and according to the endometrial molecular subgroup (**B**). **C** Representation of a selection of the most common pathogenic somatic mutations described previously in endometrial carcinomas [26, 27]. Mutation types are colored according to the legend and the endometrial subgroups are indicated: CN copy number, MSI microsatellite instability, AEC ambiguous endometrial carcinoma. The presence of different mutation types for each EC patient were grouped for primary tumors (P) and metastatic regions (M).

A total of 40 frozen samples from these 9 patients (5 EEC, 3 SEC, and 1 AEC) were analyzed by WES to a 52× median depth (range = 30–70×), recording the somatic variants identified in each sample (Supplementary Table S1). A median of 335.4 (range = 24–1537) somatic variants and 44.3 (range = 1–213) insertions/deletions (indels) per sample were identified, with a median of 179.6 (range = 12–814) non-synonymous somatic variants (Fig. 1A and Supplementary Table S2). These results were similar to those reported in the TCGA [26]. The WES data were in accordance with the IHC studies and confirmed the molecular classification of EEC3 and EEC4 as CN-low; EEC5, EEC6 and EEC7 as MSI; and SEC1, SEC2 and SEC3 being CN-high. The AEC classification was controversial and is discussed in more detail below. As expected, samples from ECs of MSI subtype harbored significantly more (One-way ANOVA test *P* < 0.001) genetic variants (median = 835.4, range = 283–1537) than samples from ECs of CN-low (median = 226.16, range = 60–404), and CN-high subtypes (median = 63.3, range = 24–112) and the AEC case (median = 233.3, range = 196–253: Fig. 1B). No significant differences in the number of mutations (synonymous and non-synonymous) were detected between paired primary tumor and metastases samples (Supplementary Fig. 2A). Furthermore, all samples analyzed harbored mutations in more than one of the most significantly mutated genes in ECs reported by the TCGA [26, 27] (Fig. 1C), with the exception of the AEC case, which only harbored a mutation in the *ESR1* gene. Thus, the most frequently mutated genes in this series were *PIK3CA* (5/9 patients, 4 EECs, 1 SEC), *PTEN* (5/9 patients, all EECs), and as expected, *TP53* and *PPP2R1A* were mutated in the SECs (Fig. 1C).

In accordance with the WES results, we observed significantly more common SNVs shared by all the tumor regions in the CN-low subgroup (EEC3 and EEC4), which shared more than 40% of the somatic variants detected (Supplementary Fig. 2B). By contrast, the MSI subgroup shared fewer SNVs (19%), suggesting an increased ITGH in this subgroup. An intermediate ITGH was observed for the CN-high SECs, whereby the number of common somatic variants in all the samples analyzed was above 26% (SNV range = 26.8–41.2%; Supplementary Fig. 2B).

**Fig. 2:**
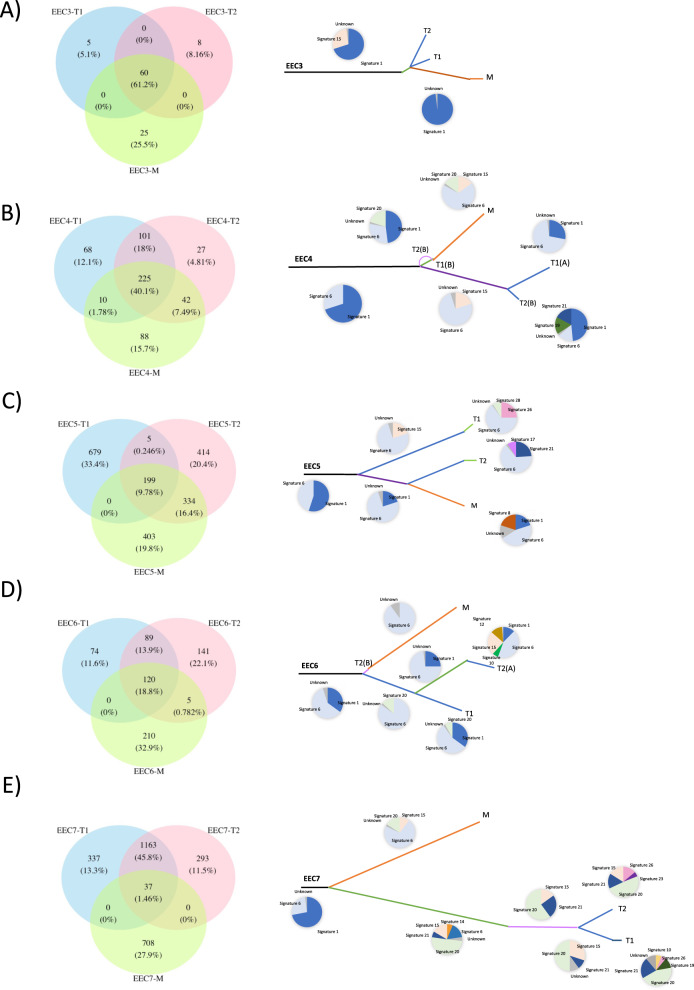
Tumor evolution of endometrial carcinomas. Venn diagrams representing the somatic mutations shared by the primary tumor sections and metastases analyzed by WES. The numbers of the genetic variants are shown, and the percentages are in brackets. Phylogenetic trees based on somatic mutations depicting the evolution of two primary tumor areas and a metastasis. The length of the branches is proportional to the number of shared or private somatic mutations. The pie charts represent the mutational signatures previously described [29] for the shared or private somatic mutations: **A** EEC3, **B** EEC4, **C** EEC5, **D** EEC6, **E** EEC7. WES whole-exome sequencing, EEC endometrioid endometrial carcinoma.

We also interrogated the mutational signatures in our series [28, 29] (Fig. 2 and Supplementary Fig. 2). All CN-low and CN-high/serous-like samples had an aging-related signature 1, whereas all MSI tumors displayed signatures 6 or 20, linked to deficient DNA MMR.

## Clonality analysis reveals a mainly monophyletic evolution of metastatic ECs

A clonal study was carried out to further assess the evolution for each tumor, which revealed important differences in the proportion of sub-clonal mutations between EECs and SECs (unpaired *t*-test, *P* = 0.02: Supplementary Fig. 2C). All except two EECs (5/7, 71%) showed less than 10% of sub-clonal variants (except for EEC3 and EEC7 with 16% and 20%, respectively), whilst SECs presented a mean of 27% sub-clonal variants. Furthermore, to gain further insight into tumor evolution, phylogenetic trees were generated based on the clusters identified by PyClone [30]. Interestingly, and as described previously [27], hierarchical clustering showed a main pattern of monophyletic evolution, with primary tumor regions more closely related among themselves than with the metastatic lesions. Metastatic lesions from several patients seem to arise from an ancestor clone (EEC3, EEC4, EEC6, EEC7, SEC1 and SEC3), with both primary and metastatic tumors also acquiring subsequent mutations (Fig. 2 and Supplementary Fig. 2D–F). In the CN-low subgroup (EEC3 and EEC4: Fig. 2A, B), a main ancestor clone represented 61.2% and 40.1% of the common variants between primary tumor (T1 and T2) and metastatic (M) regions, respectively. Of note, different regions of CN-low tumors were very similar among themselves.

By contrast, EEC5 (Fig. 2C) followed a polyphyly evolution, being the metastatic sample arising from a specific tumor region. In this case, just 9.78% of the variants were common to the T1, T2 and M regions, characterized by signatures 1 and 6. In the course of tumor evolution, the metastatic region was phylogenetically closer to T2, sharing a subset of 334 variants (16.4%), in contrast to T1 and M, in which none of the variants were exclusively shared by them. Indeed, the primary T1 tumor region acquired different exclusive mutations (33.4%), with an increase in the representation of signature 26 in the final mutations gained (also related to defective DNA MMR pathway).

Despite the polyphyly evident in EEC5, the EEC6 MSI patient (Fig. 2D) exhibited a monophyletic evolution in which the main ancestral clone contributed 18.8% of the common variants between the T1 or T2 and the M regions. Regarding the mutations identified, 32.9% of them were exclusively acquired in the metastatic region, while 11.6 % and 22.1% were acquired separately by the primary T1 and T2 regions, respectively. A distinct progression was observed in EEC7 (Fig. 2E), in which the ancestor clone only shared 1.46% of the variants in the three tumor regions. In this sub-clone, the primary tumor regions, and the metastatic lesion (a recurrent disease diagnosed 7 years after the primary tumor) followed a differential progression, with 45.8% of the mutations identified coinciding in the T1 and T2 regions. Mutational signatures suggest high diversity in their tumor evolution, being signature 20 the main one in primary tumor samples and signature 6 that of the metastasis (both related to DNA MMR deficiency) [28]. These results might reflect the early progression of an initial tumor sub-clone with stem properties that would have given rise to the recurrent disease. However, additional regions from this patient should be analyzed to confirm the distinct progression observed in this case. Alternatively, this recurrent disease could reflect clonal reduction caused by the received treatment. Nevertheless, no results relative to therapy could be extracted due to all patients received the conventional Carboplatin-Taxol therapy, therefore further studies should be addressed to explore this issue.

Regarding the serous tumors, phylogenetic analysis also revealed a monophyly evolution and in all cases, the number of variants shared by all the regions analyzed was above 20%, indicative of lower levels of ITGH. In SEC1 (Supplementary Fig. 2D), 26.8% of the variants identified in the ancestor clone were shared by the metastatic and primary tumor regions. During tumor evolution, the metastatic region retained 7.04% of the genetic variants found and primary tumor regions were grouped in a main branch. SEC2 (Supplementary Fig. 2E) was composed of a major clone in which the 30.8% of the variants were common to the three primary tumor regions and the metastatic region. From this clone, each tumor lesion evolved and acquired a specific mutational pattern, all of which were enriched for C > T transitions in the NpCpG context, consistent with signature 1 and associated with aging [28]. Similar results were obtained for the third SEC (SEC3, Supplementary Fig. 2F), which presented the strongest similarity in the four tumor regions analyzed (41.2% of common variants between T1, T2, M1 and M2).

Notably, we obtained a landscape of the EC mutation profile from the WES analysis and the evolution of the regions analyzed. Moreover, WES allowed us to identify the molecular subtypes in each sample, their associated mutational signatures, and their temporal evolution. However, the low sequencing depth in this study and the small proportion of tumor samples impaired the detection of low-frequency mutations that often account for the majority of the ITGH in a cancer. Moreover, WES studies may produce confounding results by overestimating the ITGH [18]. Thus, the mutational landscape defined in the WES study could help to select the most interesting variations in a tumor, although it will be necessary to further validate these through high-depth amplicon sequencing to overcome these problems and quantify the true ITGH.

## Decoding the ITGH in EC by targeted massively parallel sequencing

AmpliSeq analysis was performed to validate the mutations identified by WES and to perform a more detailed characterization of the heterogeneity among the samples from the same patients. Targeted massively parallel sequencing was performed on all samples analyzed by WES and additional 41 formalin fixed paraffin embedded (FFPE) samples (20 from metastasis and 21 from primary tumor) (Supplementary Table S3). A total of 532 variants were reanalyzed in the validation process (Supplementary Table S4). The selection criteria included all the pathogenic variants along with the additional non-pathogenic variants, reaching at least 40% of those detected by WES in the CN-low, CN-high and AEC case, and 10% in the MSI ECs.

Among the variants included in the validation study, we compared the proportions of somatic mutations shared by all the samples in the WES study relative to the validation targets (Supplementary Fig. 3A). For the CN-low EC, the CN-high SECs and the AEC, the number of shared variants detected in the targeted sequencing was similar or fewer than the common variants in the WES study (Supplementary Fig. 3A). For the MSI ECs, an increase in the shared variants in the targeted sequencing was found relative to the WES analyses (Supplementary Fig. 3A). Indeed, the ITGH rate was less pronounced in the targeted sequencing analysis for the MSI tumors. Probably, this is due to the fact that the sequencing depth was significantly higher in targeted sequencing than in the WES analysis (more than 20-fold) even allowing to better capture the low-frequency variants detected in the WES study.

**Fig. 3:**
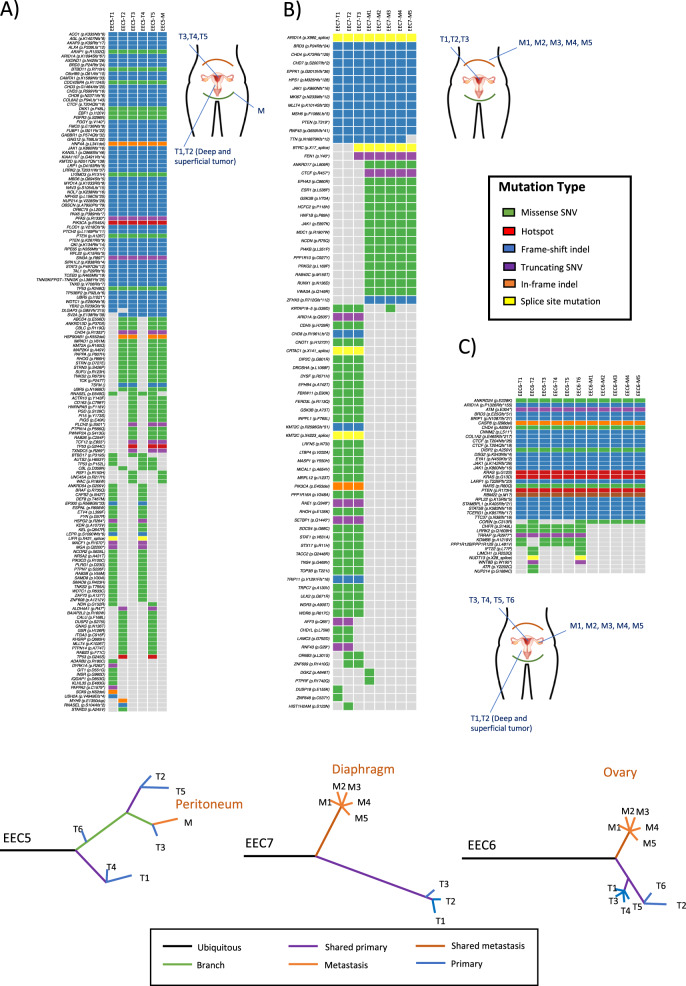
Targeted sequencing validation for MSI endometrial carcinomas. Validation of the pathogenic somatic mutations identified by targeted sequencing in primary tumor regions (T) and metastatic regions (M) of **A** EEC5, **B** EEC7 and **C** EEC6. The mutation subtypes are colored according to the legend and the origin of the tumor sections is indicated in the graphical representation: green line represents the peritoneum and orange line the diaphragm. Below each case the phylogenetic trees generated according to the presence or absence of the somatic mutations detected in the validation analysis are shown. MSI microsatellite instability, EEC endometrioid endometrial carcinoma.

The MSI ECs exhibited higher levels of ITGH among the regions analyzed and the targeted sequencing provided more information due to the improved depth of coverage. In case EEC5, only 17,93% (33/184) of the genetic alterations selected for validation were found in all the samples analyzed by WES (Supplementary Fig. 3A). However, this percentage increased in the validation study, with 35,8% of the variants found in all the samples analyzed by targeted sequencing. This increase highlights the potential of the targeted sequencing to detect low-frequency variants that were missed by WES. Indeed, 13.6% of the variants included in the validation study were restricted to the primary tumor samples by WES (identified in EEC5 T1 and/or T2), yet they were subsequently found in the metastatic regions by targeted sequencing. Despite an increase in the variants detected, the targeted validation study revealed ITGH among the different regions analyzed, as was evident through the irregular distribution of the variants identified. ITGH was even found in hotspot mutations, affecting driver genes or genes that could potentially be implicated in treatment selection, including *TP53* p.G245S and p.G244C mutations (labeled in red: Fig. 3A, Supplementary Table S4). The phylogenetic tree of this tumor reflected the ITGH, with multiple branches and sub-clones, suggesting an evolution of the metastatic sample from the primary T3 tumor region (Fig. 3A bottom), and an indication of polyphyly.

Despite the increase of genetic alterations identified in the targeted validation of EEC7, the number of shared variants was low in both the WES and the targeted sequencing (5.8% and 14.1%, respectively: Supplementary Fig. 3A). However, these results provided evidence of the tumor evolution, since the metastatic regions appeared to be developed from a common ancestral clone with the primary tumor, displaying private metastatic mutations such as those in the *RUNX1*, *ESR1* and *JAK1* genes (20% of the validated variants were exclusively identified in the metastatic regions, from M to M5). Thus, it can be hypothesized that metastatic lesions were dormant whereas the primary tumor continued to evolve, or a possible clonal selection after primary tumor treatment occurred, in accordance with the phylogenetic tree (Fig. 3B bottom). In fact, 43.3% of the validated variants were exclusively identified in the primary tumor regions (T1, T2 and T3), suggesting that they were irrelevant for its metastatic dissemination. Among the most remarkable variations observed, the primary tumor regions carried mutations in *ARID1A* and an indel in *PIK3CA* (p. E453del: Fig. 3B, Supplementary Table S4).

The mutational profile of the 12 samples analyzed from the MSI case ECC6 was very consistent across. Common variants of *PTEN*, *ARID1A*, *KRAS* and *CTCF* were identified in all regions analyzed, which shared 64.1% of the variants analyzed by targeted sequencing (Supplementary Fig. 3A, Supplementary Table S4), although more heterogeneity was found among the primary tumor samples from different regions. This uniformity was consistent with the low levels of ITGH observed among the tumor regions analyzed (Fig. 3C). In this case, two main branches were found in the phylogenetic tree, distinguishing between the primary tumor and metastatic regions in a monophyletic progression of the tumor (Fig. 3C bottom).

**Fig. 4:**
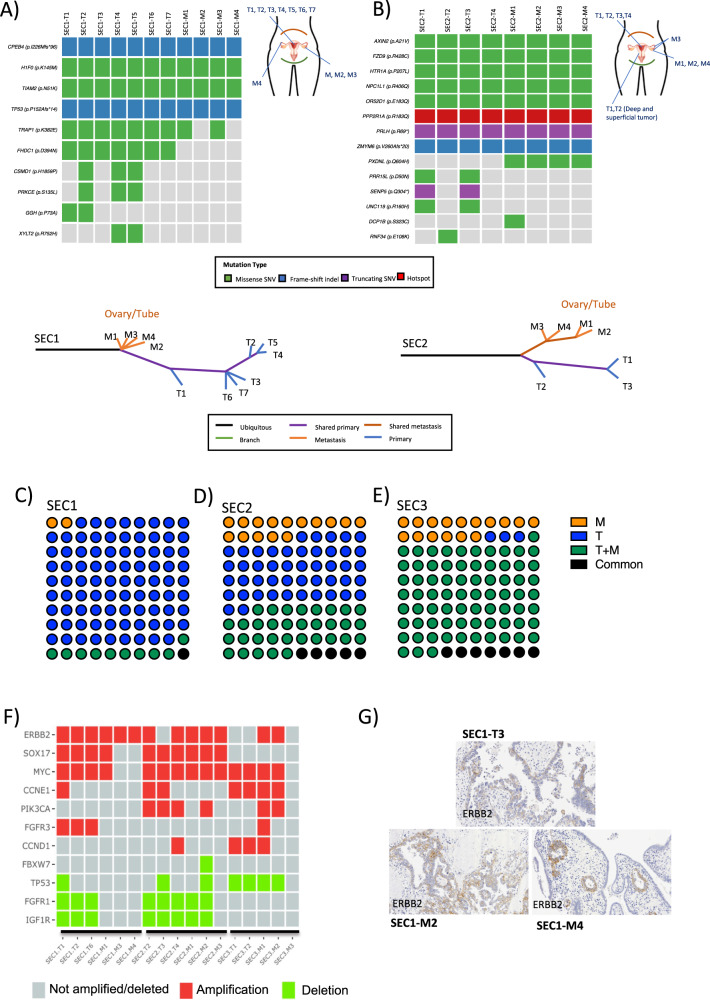
Targeted validation and Copy Number Aberrations for serous endometrial carcinomas. Validation of pathogenic somatic mutations identified by targeted sequencing in primary tumor regions (T) and metastatic regions (M) of **A** SEC1 and **B** SEC2. The mutation subtypes are colored according to the legend and the origin of the tumor sections is indicated in the graphical representation. The phylogenetic tree generated according to the presence or absence of the somatic mutations detected in the validation analysis for each case is represented below. Dot graphs represent the proportion of copy number aberrations shared between primary tumor sections (T) and the metastatic regions (M) analyzed by aCGH of **C** SEC1, **D** SEC2 and **E** SEC3. **F** The status of the most representative genes with Copy Number Aberrations described in SECs are represented: red indicates amplification and green deletion. **G** Immunohistochemical staining for ERBB2 in the SEC1 primary tumor (T3) and metastatic (M2 and M4) sections. SEC serous endometrial carcinoma. Magnification ×20.

Low ITGH was observed for CN-high SECs (Fig. 4 and Supplementary Fig. 3B) and the CN-low ECs (Supplementary Fig. 3C, D). Most genetic variants in the SECs were shared consistently among the majority of the samples of a given case. We noted, however, that the remaining mutations detected were mainly identified in the primary tumor regions, suggesting an early spread of the metastatic clone to the ovary and the subsequent development of the primary tumor in the uterus (Fig. 4A, B and Supplementary Fig. 3B). Thus, in SEC1, a group of mutations were mostly identified in the primary tumor regions (Fig. 4A). A private mutation in the *DCP1B* gene of the metastasis M1 region and a metastasis exclusive mutation in *PXDNL* were identified in SEC2 (Fig. 4B, Supplementary Table S4). In SEC3, a mutation in *TRIM27* was restricted to M2 (Supplementary Fig. 3B, Supplementary Table S4). Together, these results suggest a clonal independent synchronous evolution for the primary and metastatic regions of SECs. However, the time of sample collection was the same for primary tumor and metastasis regions and this represents a limitation, being required the analysis of additional samples to confirm the trend. Regarding CN-low cases, more than 70% of the variants were identified in the primary tumor and metastatic samples, (Supplementary Fig. 3C, D, Supplementary Table S4). In both cases, the metastatic regions analyzed belonged to a recurrent disease, affecting lymph nodes and the peritoneum in EEC3 and EEC4 patients, respectively. This is reflected in the phylogenetic trees with a long branch of ubiquitous shared variants and a final expansion of the metastatic regions due to the acquisition of additional mutations (Supplementary Fig. 3C, D bottom).

## High somatic copy number alterations ITGH in SECs

In contrast to the somatic mutations, widespread heterogeneity of somatic copy number alterations (SCNAs), defined by aCGH analysis, was found in the SECs (Fig. 4C and Supplementary Fig. 4). While 26.8% of the somatic mutations were common between the five samples of SEC1, only 1% (54/6,700) of the genes affected by SCNAs were shared across the six samples analyzed by aCGH. The three primary tumor regions harbored 87% (5856/6700) of the SCNAs identified, yet only 2% (131/6700) were detected in the three metastatic regions, indicating higher levels of heterogeneity at the SCNA level in the primary tumor regions than in metastases (Fig. 4C). It is worth mentioning the higher number of SCNAs detected in SEC2 (11,838), being 39% of SCNAs identified in one of the primary tumor regions analyzed (SEC2, T4). Increased ITGH was observed at the SCNA level in SEC2, where only 5% of the alterations identified were common between all the tumor regions. Moreover, the metastasis and primary tumor tissue had a high number of SCNAs, with 15.5% and 46.7% of the alterations observed, respectively (Fig. 4D). Similar results were observed for SEC3, where 7.4% (364/4914) of the SCNAs detected were common in all the regions analyzed and although the primary tumor regions had few SCNAs (2.8%), the metastatic regions contained 15.5% of the SCNAs identified (Fig. 4E).

Thus, SCNAs were not uniformly distributed in the regions of the SEC tumors analyzed, suggesting the presence of ITGH in each tumor. Mutations in *TP53* tumor suppressor gene and/or stabilization of the p53 protein are the most frequent alterations in SECs [31]. Although SEC3 did not harbor a *TP53* mutation (Fig. 1C), we observed *TP53* deletions in 4/5 SEC3 regions analyzed (Fig. 4F), consistent with the aberrant p53. Moreover, *TP53* deletions were detected in some of the tumor regions of cases SEC1 and SEC2 analyzed. In addition to *TP53* alterations, other focal deletions in *FBXW7, FGFR1* and *IGF1R* genes were detected [26, 32, 33]. Amplifications of the *MYC*, *ERBB2, CCNE1* and *PIK3CA* oncogenes, and of other genes like *FGFR3* and *SOX17* were also observed in some EC samples (Fig. 4F), as previously reported in ECs [26]. Of note, the *ERBB2* (*HER2*) amplification was validated in SEC1 at the protein level, in which HER2 membranous expression by IHC (2+) was seen in the primary tumor region, being even more intense in both the metastatic sections analyzed (Fig. 4G), suggesting anti-HER2 therapy as a potential treatment option in this patient.

### AEC characterization for personalized treatment

#### WES revealed ATM mutations in all AEC tumor regions analyzed

Finally, we focused on the genomic profile of the AEC characterized by a solid arrangement and lack of any kind of differentiation (endometrioid, serous, clear cell), or features of undifferentiated carcinoma (Fig. 6A, Supplementary Fig. 1A). The WES study of this tumor identified molecular features completely different to the other EC samples. Despite the identification of 85 likely pathogenic somatic variants (Supplementary Table S1), none of these involved genes were commonly altered in EC and SEC subtypes. Instead, we identified a pathogenic mutation in all AEC tumor regions analyzed which affected a splice site of the *Ataxia Telangiectasia Mutated* gene (ATM, c.6096-1 G > A) and was associated with loss-of-heterozygosity (LOH).

**Fig. 5:**
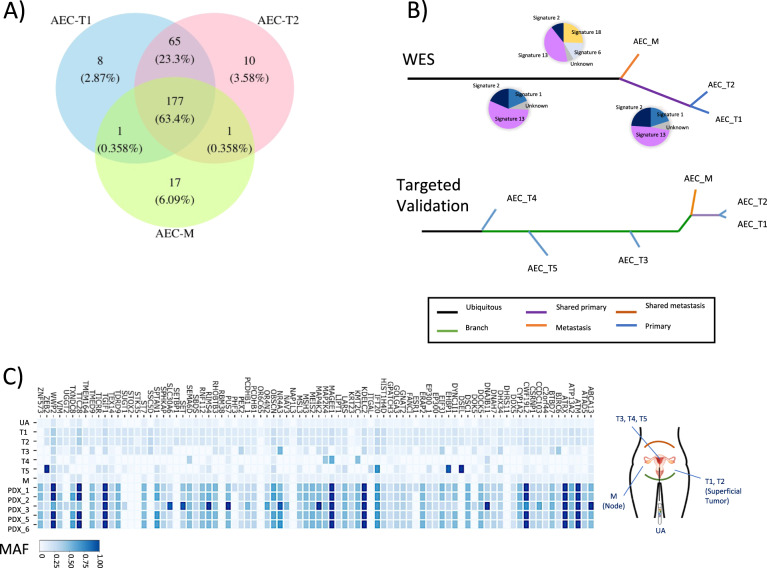
Analysis of the ambiguous endometrial carcinoma and personalized treatment. **A** Venn diagram representing the genetic variant distribution in two areas of the primary tumor (T1 and T2) and in the lymph node metastasis (M) of the ambiguous endometrial carcinoma (AEC) identified by whole-exome sequencing (WES) analysis. The numbers of genetic variants are shown with their percentage in brackets. **B** Phylogenetic tree based on somatic mutations depicting the evolution of the primary tumor areas and the metastatic regions, and a representation of the molecular signature obtained by the whole-exome sequencing (WES) (top) or targeted validation (bottom), color coded according to the legend. **C** Mutation Allele Frequency (MAF) heat-map based on the targeted sequencing validation of somatic mutations in multiple primary and metastatic samples from the ambiguous endometrial cancer and patient-derived xenograft (PDX) models. Tumor locations: T1–T5 different uterine locations of the primary tumor, M lymph node metastasis—detected and resected 7 years after primary tumor diagnosis, UA uterine aspirate obtained at time of the surgery.

The proportion of somatic mutations shared between the two primary and the metastatic areas was high, with more than 63% of the genetic alterations being present in all the samples analyzed (Fig. 5A). The phylogenetic tree based on the somatic mutations had two main branches, one corresponding to the primary tumor samples and the other to the metastatic sample. A monophyletic evolution (Fig. 5B) similar to the majority of ECs was observed, although its mutational profile was distinct from that of the remaining ECs analyzed. Despite the ambiguous features identified in the AEC case, the patient showed reduced survival rates, similar to SEC (Supplementary Fig. 1D). Interestingly, mutational signatures associated with the AID/APOBEC family of cytidine deaminases (i.e., signatures 2 and 13 [34]: were detected in all AECs lesions analyzed. The APOBEC mutational signature has mainly been reported in bladder, breast, cervical and head and neck cancers [28, 35–39], but also defects in DNA repair mechanisms including APOBEC have been described as a potential players in the progression from primary to metastasis in EC [40, 41].

### Targeted validation of the AEC, including uterine aspirates and PDX

Like the other ECs, targeted sequencing was used to validate the AEC genetic variants found in the WES study, validating 86 of 215 variants tested (40%) by Ampliseq sequencing (Supplementary Table S4 and Fig. 5B bottom). In addition to the samples analyzed by WES, three FFPE areas of the primary tumor (T3, T4 and T5), as well as a uterine aspirate (UA) obtained at time of the surgery, were also analyzed (Supplementary Table S3). In the validation process, only 17% of the variants were found to be common between all samples (T1-T5, M and UA) compared to 69% of the selected variants from WES (Supplementary Fig. S3A). Despite the differences observed among the tumor regions, metastasis, and UA, T1 and T2 shared the 96.4% of the mutations analyzed and T1-T3 and T2-T3 the 69.4% for each case (Fig. 5C). However, this proportion was lower (<47%) between these three regions and T4 or T5, which could be related to the purity of primary T4 and T5 regions [42]. This patient’s tumor had a polyphyletic evolution (Fig. 5B), whereby all the samples seemed to evolve from a single truncal clone, with the primary T1 and T2 tumor regions, and the M lesion having a longer evolutionary process than T3, T4 and T5, as reflected by an accumulation of mutations restricted to these samples. These results were reflected in the large percentage (77.9%) of mutations shared among T1, T2 and M. Furthermore, 82% of the variants analyzed were detected in the UA sample, which also contained 22.8% of the somatic mutations detected in the metastatic region and those were not found in T1, T2 and T3 (T4 and T5 were discarded due to low purity: Fig. 5C). Although low levels of metastatic mutations were detected in the UA, this sample has the capacity to capture some of them in a non-invasive way and recapitulate an important part of the ITGH in EC [13].

**Fig. 6:**
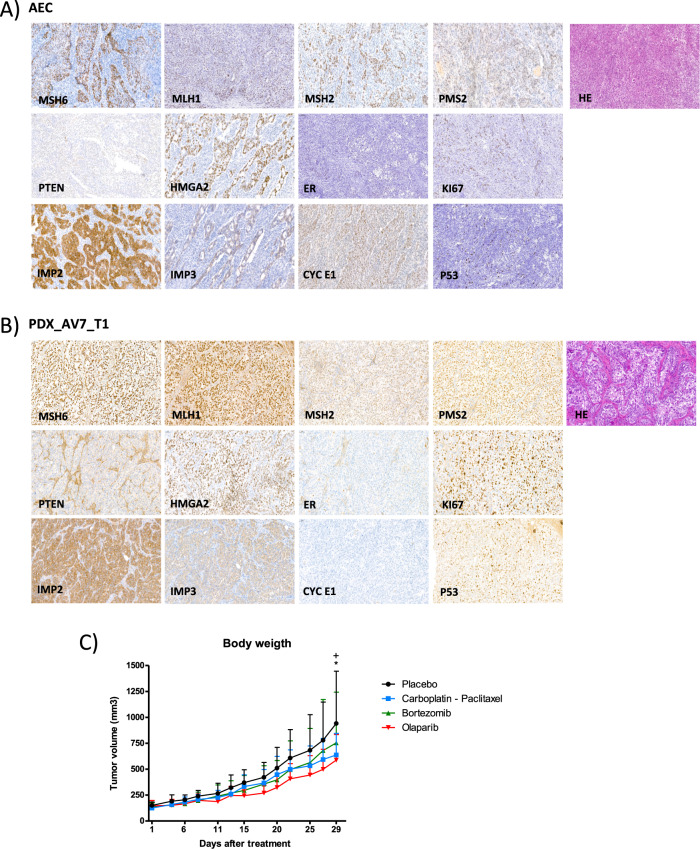
Histological and immunohistochemical profiles for the ambiguous endometrial carcinoma. Histological (HE) and immunophenotype of the ambiguous endometrial carcinoma based on a set of proteins described previously to help discriminate between SEC and differentiated EECs: MSH6, MLH1, MSH2, PMS2, PTEN, HMGA2, ER, ki67, IMP2, IMP3, CYC E1 and p53 [24] in AEC patient (**A**) and PDX derived from tumor 1 (**B**). Magnification ×20. **C** Growth of AEC-derived PDX tumors treated with the drugs indicated for 30 days. Tumor volumes were measured three times each week with a digital caliper and the volumes were calculated as: (length × width^2^)/2 = mm^3^; *p-value < 0.05, significantly different placebo vs. Carboplatin-Paclitaxel; +*p*-value < 0.05, significantly different placebo vs. Olaparib analyzed by one-way ANOVA. The data show the mean of 6–7 independent mice for each treatment.

In parallel, we developed PDXs from the AEC as a preclinical model to gain insights into tumor evolution and the impact of therapies. A total of 5 different PDXs were set up from the AEC by implanting five different tumor samples into distinct mice, samples from: the superficial region of the tumor (AEC_PDX1); the deepest part of the tumor at the myometrial invasion front (AEC_PDX2); the right lymphatic node metastasis (AEC_PDX3); the left lymphatic node metastasis (AEC_PDX4); and the tumor infiltrating the cervix (AEC_PDX5). After tumor growth, the PDXs were resected and analyzed with a panel of biomarkers previously used to differentiate between high-grade endometrioid and serous carcinoma [24]. As previously observed in the AEC patient, the panel of biomarkers employed for the analysis of the PDXs (two PDXs showed as a representation in Fig. 6B and Supplementary Fig. 5A) showed features from both histological subtypes, with some biomarkers indicating a serous pattern while others an endometrioid one. The five PDX tumor samples were analyzed by targeted sequencing, which detected 79 of the 86 variants analyzed for the AEC patient (81%: Fig. 5B bottom). In addition, a WES analysis was also performed on the PDXs and in normal mouse tissue as a control, identifying a total of 395 variants (Supplementary Table S5). Interestingly, only 175 of the 395 (44%) variants had previously been identified in the patient’s samples, most of them were common between all the PDX samples (166/175, 95%). A total of 83 of the 258 variants (32%) previously detected in the patient’s WES analysis were not identified in the PDXs (Supplementary Fig. 5B). These findings suggested that PDXs harbored most of the variants previously identified in the respective human tumor, primarily those that were present in all the patient samples. Accordingly, the PDX models were potentially derived from sub-clones of the tumor of origin, which might underrepresent the ITGH found in the different samples of the original tumor, and indeed, the PDXs may undergo additional evolution following implantation.

#### Olaparib as a potential treatment for the AEC patient

Finally, to identify new therapeutic approaches for the AEC patient, we used the molecular profile obtained for this patient to carry out an in silico analysis in different drug databases. The results produced high scores for drugs classified as inhibitors of nucleic acid synthesis, such as poly (ADP) ribose polymerase (PARP) inhibitors [43] (Supplementary Table S6) and drugs like Bortezomib and Paclitaxel. All samples analyzed by WES harbored pathogenic mutations in the *ATM* gene associated with LOH (Supplementary Table S1). In this regard, there have been reports of clinical benefits when the PARP inhibitor Olaparib has been used to treat cancer patients with somatic *ATM* mutations [44–46]. Thus, we assessed the benefits of treatment with Olaparib and Bortezomib relative to that of the standard EC treatment, carboplatin-paclitaxel [47, 48], in the F2 generation of the AEC_PDX1. A significant therapeutic effect was attributed to the Olaparib treatment, which reduced tumor growth relative to the placebo (*p*-value < 0.05: Fig. 6C). Together, these data support the feasibility of using genomic studies to identify new therapeutic opportunities, especially in cases of aggressive and/or ambiguous tumors.

## Discussion

ITGH is a consequence of deficient DNA replication that has been described since the early days of cancer research [49–51]. Genetic differences not only exist between diverse cancers but also, within the same tumor [52]. The identification of cancer-driver mutations and genomic alterations in tumors from different patients, and within individual tumors, establishes the basis for precision medicine, driving the treatment of the patient according to the genetic alterations present in their tumors. The development of high-throughput techniques and specifically, massively parallel sequencing, has made a real difference in our understanding of ITGH.

Numerous studies have shed light on the extent of tumor diversity in distinct solid tumor types, such as pancreatic [53, 54], lung [55, 56], breast [57–59], colorectal [60, 61] and prostate tumors [62, 63]. Similar analyses have been also carried out on gynecological cancers [64], mostly focusing on ovarian carcinomas [65, 66]. However, there are other tumor subtypes that have been less well explored, as it is the case of EC. Here, at least two regions from primary EC tumors and one from a metastatic lesion were analyzed by WES, revealing that less than half of the mutations were shared between multiple samples from each EC patient, in line with results obtained for other tumor types [67, 68]. However, this differs from the homogeneity found in pancreatic or lung carcinomas, where around 70% of the variants are ubiquitously detected [69–71]. For tumors that can be completely resected, the sequencing of a single region is generally adequate to select targeted therapies, however multi-region sequencing is critical to evaluate the potential targeted-therapies that could be effective when tumors cannot be completely removed and therefore, the majority of mutations are unlikely to be represented [52].

Here, different degrees of ITGH were found between the multiple EC molecular subgroups, the highest number of mutations identified for the MSI subgroup, coupled with the highest level of ITGH. This ITGH was less pronounced for the CN-high/SEC subgroup (30% common SNVs) followed by the AEC patient (40% common SNV), being the lowest levels of ITGH found in the CN-low ECs, where more than 60% of the variants were shared among all the regions. Furthermore, the majority of variants detected were associated with a clonal status in each sample, suggesting that most of the cancer cells carried the majority of variants detected in this specific region when a single sample was considered. This could be misunderstood as the absence of heterogeneity, however, clonal mutations in one region of the tumor could be completely absent in another tumor region, as reported previously for renal [10], lung [70] or ovarian [65] cancers. Regarding tumor progression, a high proportion of monophyletic evolution has been described in EC [27]. Indeed, we observed monophyly in 7 of the 9 patients and polyphyletic evolution in the remaining two cases (EEC5 and SEC2), although this earlier study did not examine multiple regions from the same primary tumor [27].

The low sequencing depth of the WES and the low tumor proportion sampled represent the most important limitations in our study. WES studies provide the mutational landscape of the EC samples, the evolution of the regions analyzed and identify the molecular subtypes. Although our WES study provided evidence of the presence of ITGH in EC patients, there was more limited detection of low-frequency mutations which often include private mutations that represent the majority of the ITGH within a cancer [18]. To address this issue, pathogenic/recurrent variants identified in the WES were further analyzed in the validation study, which included additional tumor samples. Interestingly, as well as the common mutations found in each tumor, more specific variants were mainly identified in the primary tumor regions in comparison with the metastatic lesions.

It is interesting to compare the metastatic timeline of EC to different organs based on the phylogenetic trees. Peritoneal and lymph node metastasis are expected to occur late in the process of tumor dissemination, as observed in the trees for EEC3, EEC4 and EEC5. However, the timeline for ovarian metastasis is probably more variable, in particular for EEC. There is a subset of patients with EEC and ovarian metastasis that have a very good prognosis. In fact, historically patients in this type of tumors were interpreted as having two independent primary tumors due to their indolent behavior [72–76]. However, mutation analysis suggested that both tumors have a clonal origin and thus, ovarian lesions should be considered as metastases [77–80]. Several hypotheses have been proposed to explain such a favorable prognosis. The most feasible explanation is that in this group of patients, ovarian metastases occur through fallopian tube migration at early stages of tumor progression. By contrast, endometrioid carcinomas with ovarian metastasis and a poor prognosis would disseminate though lymphovascular invasion, and this metastatic spread would occur late in the process of tumor progression. Nevertheless, in our series of cases, patients with ovarian metastasis had a worse prognosis and died from their disease irrespective of their molecular subgroup (EEC6, SEC1 and SEC3). The phylogenetic trees of these tumors had a similar form, in which metastasis occurred early in tumor progression. These results could reflect how the metastatic disease appears from an initial clone of the primary tumor with specific molecular features that then spreads to the ovary, while the same tumor clone in the uterus would continue acquiring mutations over time, modifying the initial tumor profile. Nevertheless, the number of samples with this pattern analyzed in our study is limited and more samples should be included to extract definitive conclusions. A better prognosis and the same tumor evolution were observed for SEC2, which also developed an ovarian metastasis, and for the ECC7 with a diaphragm metastasis, being both patients alive and disease-free. Recognition that ovarian metastasis occurs early in tumor dynamics in a subset of our patients supports the idea that the timeline of metastasis may be related to the prognosis in these patients. However, additional tumors should be analyzed in order to determine if this pattern might be related to tumor histology or to metastatic tropism.

Conversely, EEC3 and EEC4 patients had more variants in their metastatic lesions, lymph nodes and omentum than in the primary tumor regions. In both cases, metastases were detected as a recurrent disease after 3 years of primary tumor treatment. These results may indicate that recurrent metastatic samples acquire additional mutations during or after tumor treatment, which favored their growth even after years of disease remission. In these cases, metastasis would slowly acquire a more complex genetic phenotype, which could lead to an aggressive behavior during their development.

Despite the low levels of ITGH observed in SECs, both in the WES and targeted sequencing studies, CGH studies highlighted ITGH at the SCNA level among different tumor regions from the same patient for the most important genes affected by SCNA in EC [26]. In this molecular subgroup, amplifications and/or deletions were observed in the primary and metastatic regions, with neither significant differences among them nor clear evidence of metastasis-specific exome mutations, as reported previously for EC and other cancers [27, 81]. Thus, the mutation pattern does not fulfill an essential role in the development of metastasis, or there are no common genetic mechanisms to generate them in ECs. It is also important to note that other genomic alterations like chromothripsis or important genomic changes could participate in metastasis, as observed in other cancers [82]. *ERBB2* amplification was detected in the CGH study for SEC1, SEC2 (both the primary tumor and metastasis) and SEC3 (only in metastasis), while the IHC analysis of different tumor regions reflects the ITGH in the patient and could reveal new targets for available treatments.

Regarding the mutational profile of AEC, we determined and confirmed the absence of the most frequent molecular alterations in EC by two different approaches. AEC analysis revealed a mutational landscape not closely related to either high-grade ECs or SECs. AEC is an uncommon type of cancer, previously reported in the literature [5–7] and difficult to classify at the morphological and molecular level. The diagnosis of AEC is hard to made in the absence of an intense molecular analysis, as was performed in our case. AEC cases are occasionally placed in the spectrum of high-grade endometrioid carcinomas, although they are very aggressive, and do not fit perfectly in any TCGA subgroup. For these reasons, it is important to be aware of the existence of this unusual type of tumor. Lower levels of ITGH were detected in AEC, in which there was a stronger similarity between the two primary tumors and the metastatic lesion. These findings suggest a monophyletic evolution of this tumor, whereby the primary and metastatic lesions arose from an ancestral clone. Interestingly, signatures associated with the AID/APOBEC family were associated with the regions analyzed in the AEC, although these signatures are less frequent in EC [40, 41]. However, these signatures have been correlated with immune cell populations modulating the immunological response [83–85].

Despite the decreased ITGH observed in our WES studies, this was stronger when additional tumor regions were studied by targeted sequencing, highlighting the need for including different tumor sections to better characterize the tumor at the molecular level. Remarkably, our results confirm that UAs could recapitulate the ITGH in EC in a best way than traditional biopsies [13]. Furthermore, all the samples from the AEC patient analyzed by WES had only one pathogenic variant that affected a splice acceptor site in the *ATM* gene, as validated by massive parallel targeted sequencing. This gene is involved in DNA repair and commonly found mutated in cancers associated with poor outcomes and resistance to chemotherapy [86]. Consistent with previous observations demonstrating that *ATM* and *TP53* loss of function mutations are mutually exclusive [87], this high-grade AEC lacked *TP53* mutations, yet it harbored bi-allelic *ATM* inactivation. To further investigate the biology of this AEC, five PDXs were set up from the superficial and deepest primary tumor areas, as well as from metastatic foci. Targeted massive parallel sequencing revealed that most of the mutations previously detected in the patient (81%) were also found in all the PDXs analyzed, including that found in the *ATM* gene. This did not occur with most of the variants that were heterogeneously identified in the patient, which were not observed in the PDX models. Moreover, a personalized treatment for the AEC patient was explored, being PARP inhibitor considered a new potential therapeutic approach in this specific tumor context. The PARP inhibitor Olaparib has a good response rate in patients with *ATM* mutations [44–46] and it significantly controlled tumor growth in the AEC PDX models. Overall, our findings suggest that Olaparib could be considered a potential treatment for this AEC patient. Nevertheless, additional studies should evaluate if other EC patients with *ATM* mutations could benefit from therapy with Olaparib.

**Fig. 7:**
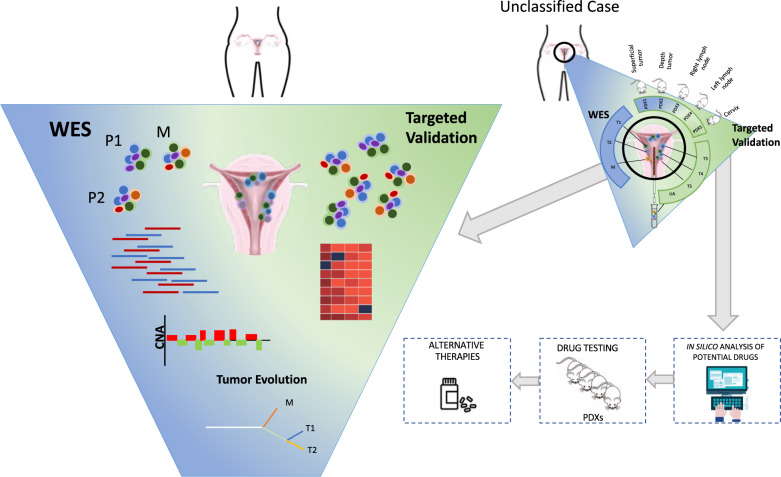
Summary of the procedure followed for samples included in the study. WES whole exome sequencing, P primary tumor, M metastasis, CNA copy number aberrations, PDX patient-derived xenografts.

Taken together, although our study presents some constraints in the number of analyzed patients by subtype to extract outright conclusions and more samples should be studied to resolve these limitation, a mean of 9 sample regions per tumor have been analyzed which should be considered an important number to infer the ITGH. Furthermore, this study emphasizes the significance of analyzing the maximum number of samples to better understand the tumor evolution, especially when the genetic heterogeneity found between multiple samples of the primary tumor is considered (Fig. 7). Nevertheless, it is not useful for current management strategies, and it is fairly difficult to calculate the number of tumor regions needed to ensure proper assessment of ITGH, not least as it is not possible to anticipate the degree of ITGH for each tumor. Another relevant issue is the sequencing methodology used in the ITGH study. Our study presents an important limitation of WES sequencing depth and in the percentage of the cancer cell fraction (CCF), which could overestimate the ITGH found. However, deeper validation sequencing on a different platform and the use of additional samples can mitigate this problem and permit ITGH quantification. Although several studies have suggested that ITGH could be an independent prognostic factor of disease progression and survival in other types of tumors [88], the mechanisms underlying this phenomenon remain unclear. The fact that some ECs show resistance to treatment has been related to the hypothetical ITGH of the primary tumor [89]. Indeed, reconstruction of the phylogenetic tree reflects how tumor evolution could be correlated with tumor progression, prognosis, and the presence of a recurrent disease (Fig. 7). Thus, our study takes into account the relevance of studying different tumor regions by WES, for capturing the ITGH in EC patients with controversial clinicopathological features, such as AEC, and could assist to spot the most appropriate treatment set up.

## Materials and methods

### Human samples

A series of 9 metastatic EC cases for which multiple fresh-frozen tumor zones were available were collected from the Arnau de Vilanova University Hospital (Lleida), the MD Anderson Cancer Center (Madrid), the Vall d’Hebron University Hospital (Barcelona) and the Bellvitge University Hospital (Barcelona), with the support of the MD Anderson Foundation Biobank (record number B.0000745, ISCIII National Biobank Record), the Xarxa Catalana de Bancs de Tumors and Plataforma de Biobancos ISCIII (PT13/0010/0014, B.000609: CEIC PR(AMI)364/2014). In addition, a uterine aspirate (UA), a minimally invasive sample of the endometrium collected for diagnostic purposes, was obtained from the AEC patient and used in the validation study (Table 1). The patient samples were selected following the study criteria, and the procedures established in current Spanish Law 14/2007 on Biomedical Research and the 1716/2011 Royal Decree on Biobanks. The local ethical committee from each institution approved the study and complete written informed consent was obtained from all the patients. The histological classification was performed by two pathologists (ARS, XMG), determining the subtype according to the World Health Organization (WHO) criteria [90]. The International Federation of Gynecology and Obstetrics (FIGO) guidelines [91] were used for the staging and grading of each tumor.

For each samples analysis DNA extraction, the Promise TCGA molecular classification of EC patients, IHC analysis, Array Comparative Genome Hybridization (aCGH) methodology and TumorTracer Server have been detailed in Supplementary Material and Methods

### Whole-exome sequencing

WES was performed in collaboration with Sistemas Genómicos, S.L. (Paterna, Valencia, Spain) and the MSKCC Integrated Genomics Operation (NY, USA). Fresh-frozen regions were analyzed by WES using the SureSelectXT Human All Exon + UTR (71 Mb) v5 enrichment kit (Agilent, Santa Clara, CA) and paired-end sequencing was performed in an Illumina HiSeq 2500 sequencer (Illumina, San Diego, CA). A median depth of coverage was achieved of 51x (range = 32–74×) for tumor samples and 52× (range = 45–60×) for normal samples. A total of 40 frozen tissue samples analyzed by WES were subjected to Ampliseq targeted re-sequencing to validate the variants selected. The Targeted resequencing analysis has been included in Supplementary Material and Methods.

### Bioinformatics analysis

The bioinformatics analyses were performed as described previously [92, 93]. Briefly, reads from WES and targeted sequencing were aligned to the human reference genome GRCh37 using Burrows-Wheeler Aligner [94]. Somatic single nucleotide variants (SNVs) were identified using MuTect [95], whereas small insertion and deletions (indels) were identified using Strelka [96] and VarScan 2 [97], using paired tumor/normal approach to exclude germline mutations. The potential functional effect of each non-synonymous SNV was analyzed in silico as described previously [77], using a combination of multiple predictors. The CCF was calculated using ABSOLUTE and reviewed manually [26, 88, 98]. The mutational signature of each EC studied was obtained by the analyzing the mutational context, as described previously [28, 77]. Briefly, SNVs were categorized into C > A, C > G, C > T, T > A, T > C or T > G and then sub-categorized according to the nucleotides preceding (5') and succeeding (3') the mutated base. The mutational patterns found in our samples were compared to the previously published data [28], after normalization according to the frequency of nucleotide changes observed in the respective sequencing platforms.

### Phylogenetic tree generation and clonality study

Maximum parsimony phylogenetic trees were generated using CCF values from the WES analysis and the data on the presence/absence of somatic mutations in the validation of each tumor region, as described previously [77]. Briefly, the R package Phangorn was used to apply the Neighbor-joining method and to obtain the Hamming distance, optimized using the parsimony Ratchet method [99]. To further determined the clonal composition of metastatic ECs in the WES study, a Bayesian clustering model (PyClone [30]: was applied to variant allele fractions, incorporating tumor cellularity, ploidy and local copy number obtained from ABSOLUTE [98] and FACETS [100], as described previously [101].

### In silico prediction of drug treatment

Compound databases CTD (http://ctdbase.org/) and STITCH (http://stitch.embl.de/: [102–104] were used to identify potentially effective drugs according to the mutational status of genes identified in the WES carried out on the AEC patient. The drugs identified were evaluated in a preclinical study using PDX models from the AEC patient (AEC_PDX). The significance was analyzed using the non-parametric Mann–Whitney test.

### Patient-derived xenografts: generation and treatment

PDXs were performed at the Biomedical Research Group in Gynaecology (Vall d’Hebron Institute of Research) and all the procedures involving animals were performed according to protocols approved by the Animal Experimentation Ethics Committee at the Vall d’Hebron University Hospital (CEEA 16/16). Details are specified in Supplementary Material and Methods.

### Statistical analysis

The data was compared between the TCGA dataset, and the patient series included in this project using the Mann–Whitney *U* test. Patient subgroups were compared with an unpaired Student’s *t*-test (comparisons between two groups) or with one-way ANOVA and Tukey’s multiple comparison tests (comparisons between more than two groups). Statistical analyses were performed using the SPSS Statistics 17.0 software package (SPSS Inc., Chicago, IL). For SCNA comparison between WES and aCGH, “in house” python scripts were used. The concordance between both analyses was described with the kappa value using R [105]. OS was performed by Kaplan–Meier statistics using follow-up data from EC patients to analyze the prognostic value of molecular/histological subgroups. OS studies were performed using survival R package (https://CRAN.R-project.org/package=survival).

## Supplementary information

Supplementary Information

Supplementary Figures 1-6

Supplementary Table S1

Supplementary Table S2

Supplementary Table S3

Supplementary Table S4

Supplementary Table S5

Supplementary Table S6

Supplementary Table S7

## Data Availability

The datasets generated during the current study are available in the NCBI BioProject PRJNA378720 (ID: 378720) and with GEO accession number GSE101447.
