# Supplementary material for: Intratumor genetic heterogeneity and clonal evolution to decode endometrial cancer progression
Source: Oncogene. 2022 Feb 10;41(13):1835–50. doi: 10.1038/s41388-022-02221-0 (PMC8956509; doi:10.1038/s41388-022-02221-0)
Supplement: Supplementary file 1 — Supplementary Information [file 41388_2022_2221_MOESM1_ESM.docx]

Intratumor genetic heterogeneity and clonal evolution to decode endometrial cancer progression

by Mota A & Oltra SS et al.

***Index***

*Supplementary Materials and Methods 3*

*Supplementary References 6*

*Legends of Supplementary Tables and Figures 6*

***Supplementary Materials and Methods***

***DNA extraction***

DNA was extracted from several FFPE or frozen tissue sections (10-15 μm) and UA pellets. Briefly, hematoxylin and eosin (HE) stained sections of all the frozen tumors and matched normal tissue were all reviewed to ensure at least 90% tumor purity and a lack of tumor contamination in the normal samples. Genomic DNA was extracted from the frozen tissue and FFPE sections by phenol extraction and ethanol precipitation, and from peripheral blood using the DNeasy Blood and Tissue Kit (Qiagen, Germantown, MD). The DNA recovered was quantified by NanoDrop 2000 Spectrophotometer and Qubit® 3.0 Fluorometer assay (Life Technologies, Carlsbad, CA, USA) and the DNA quality was evaluated using electrophoresis in agarose gel (Agarose D1 Low EEO, Conda-Pronadisa), visualized using the Chemidoc XRS+ Molecular Image (BioRad).

***Promise TCGA molecular classification of EC patients***

ECs were classified into the molecular subtypes using the ProMiSe classification according to the TCGA classification (1). Immunohistochemistry for tumor protein 53 (p53) was performed and the present/absent of mismatch repair (MMR) proteins was analysed by MLH1, MSH2, MSH6 and PMS2 immunohistochemistry. MMR-deficient/ MSI molecular subtype ECs were identified with at least one defective mismatch repair protein (2). *POLE* hotspot mutational status was analyzed by Sanger sequencing using two different sets of primers for P286R (F: 3’- CCCATCCCAGGAGCTTACTT-5’; R: 3’- GTGTTCAGGGAGGCCTAATG-5’) and V411L hotspots (F: 3’- ATGTCCTCCGGGTCTAGCTC-5’; R: 3’- TTGCATCTGTCTGTGTGGTG-5’), and the information derived from the WES and the custom Ampliseq targeted sequencing to confirm the results.

***TumorTracer Server***

The TumorTracer server was used to correctly determine the origin of the AEC sample in terms of their molecular profile (3). The classification score was defined as the proportion of trees from the validation dataset, i.e.: the proportion of samples with similar molecular alterations to the input sample.

***HPV DNA detection***

DNA from patient sample was extracted as previously mentioned. PCR multiplex was performed for the viral genes E6 and E7 to detect high-risk oncogenes (HPVs type 16, 18, 31, 33, 35, 39, 45, 51, 52, 56, 58, 59 and 68, according to the IARC) followed by the amplification of the GP5/6 region of L1 gene by PCR. For this study we follow previous established protocols by (4).

***Immunohistochemistry***

Immunohistochemistry was performed on 2-4 μm FFPE tissue sections. After deparaffinization (60 °C for 1 hour), the PT Link module (Dako, Denmark) was used for antigen retrieval. Immunohistochemistry, amplification and visualization of immune complexes was performed in an Autostainer (Dako, Denmark), using EnVision FLEX+ (Dako, CA, USA), and all the antibodies and conditions used are included in Supplementary Table S7.

***Targeted amplicon resequencing for WES validation***

A total of 40 frozen tissue samples analyzed by WES were subjected to Ampliseq targeted re-sequencing to validate the variants selected. An additional 41 frozen and FFPE tumor regions were also sequenced to expand the study (Supplementary Table S3). A total of 532 variants detected by WES were covered by custom Ampliseq panels (Supplementary Table S4). Primers were designed using the Ion AmpliSeq™ Designer tool. Libraries were generated as described previously (5) and sequenced in the Ion PGM System (Life Technologies) according to the manufacturer’s indications to a mean depth of 1,243x (range = 224-5,431x).

***Array Comparative Genome Hybridization (aCGH)***

A high-resolution array comparative genomic hybridization (aCGH) analysis was performed on a group of SEC samples prone to have CNVs (Supplementary Table S3). An Agilent SurePrint G3 Human CGH 180K microarray (AMADID 022060 Agilent Technologies, Santa Clara, CA) that spans the entire human genome at a median resolution of 17 kb was used and Agilent Technologies standard operating procedures were followed. As such, 500 ng of genomic DNA from the primary tumor or metastatic DNA (that used previously for WES) and human reference (Agilent) genomic DNA was fragmented by dual enzymatic digestion (AluI and RsaI), and then differentially labeled by random priming with Cy5-dCTP and Cy3-dCTP, respectively. Hybridization was carried out according to the manufacturer's protocol and the arrays were then scanned on an Agilent DNA Microarray scanner G2565CA (Agilent Technologies). The microarray data were extracted and visualized using Feature Extraction software v10.7 and Agilent Genomic Workbench (AGW) software v7.0 (Agilent Technologies), and copy number altered regions were detected using the Aberration Detection Method 2 (ADM-2) algorithm set as 6, with a minimum number of three consecutive probes.

***Patient-derived xenografts: generation and treatment***

Patient derived xenografts (PDXs) were performed at the Biomedical Research Group in Gynaecology (Vall d’Hebron Institute of Research) and all the procedures involving animals were performed according to protocols approved by the Animal Experimentation Ethics Committee at the Vall d’Hebron University Hospital (CEEA 16/16). Five PDX models were established for the AEC patient through the subcutaneous implantation of xenografts from five different tumor areas into five different mice: PDXs from a region of superficial tumor (AEC_PDX1), deep tumor (AEC_PDX2), right lymphatic node (AEC_PDX3), left lymphatic node (AEC_PDX4) and cervix (AEC_PDX5). After growing in the mice, the tumors were resected, and they were then used for expansion or processed by formalin fixation and freezing. In order to validate the potential effect of drugs identified in an *in silico* study, the AEC_PDX1 graft was expanded and established in 6 week-old female Swiss nude mice. These mice (6-7 per group) were randomly assigned to the following treatment groups: vehicle, EC standard chemotherapy (20 mg/kg Carboplatin – 5mg/kg Paclitaxel, i.p. once a week), Bortezomib (1mg/kg, i.p. every 3 days) or Olaparib (50mg/kg, i.p. daily). All treatments were administered for 4 weeks, measuring the tumor sizes twice weekly with a vernier caliper and calculating the volume as length×width^2^×0.5.

***Supplementary References***

1. Cancer Genome Atlas Research N, Kandoth C, Schultz N, Cherniack AD, Akbani R, Liu Y, et al. Integrated genomic characterization of endometrial carcinoma. Nature. 2013;497(7447):67-73.

2. Buza N, Ziai J, Hui P. Mismatch repair deficiency testing in clinical practice. Expert Rev Mol Diagn. 2016;16(5):591-604.

3. Marquard AM, Birkbak NJ, Thomas CE, Favero F, Krzystanek M, Lefebvre C, et al. TumorTracer: a method to identify the tissue of origin from the somatic mutations of a tumor specimen. BMC Med Genomics. 2015;8:58.

4. Prakash P, Patne SC, Singh AK, Kumar M, Mishra MN, Gulati AK. PCR and Genotyping for HPV in Cervical Cancer Patients. J Glob Infect Dis. 2016;8(3):100-7.

5. Mota A, Colas E, Garcia-Sanz P, Campoy I, Rojo-Sebastian A, Gatius S, et al. Genetic analysis of uterine aspirates improves the diagnostic value and captures the intra-tumor heterogeneity of endometrial cancers. Mod Pathol. 2017;30(1):134-45.

***Legends of Supplementary Tables and Figures***

**Supplementary Figure S1. Immunohistochemical and genomic representations of endometrial carcinomas, and tumor tracer classification of ambiguous endometrial carcinoma. A**) The most representative biomarker for the EC subgroups: Estrogen receptor (ER) positive is representative of copy number low (CN-low), MLH1 is representative of microsatellite instability (MSI) tumors, TP53 overexpression is characteristic of copy number high (serous carcinoma, SEC), and the ambiguous endometrial carcinoma (AEC) without a clear molecular subgroup pattern. (**B**) The TumorTracer classification score identified the endometrium as the most probable tissue of origin (left). Representation of the number of tumors from the TumorTracer dataset incorrectly or correctly classified depending on the degree of confidence (right). The TumorTracer classification and confidence scores obtained for our patient are marked with a red arrow. (**C**) Detection of HPV DNA based on multiplex-PCR (left) and PCR/sequencing (right) procedures of the viral genes E6/E7 and L1, respectively. (**D**) Overall survival study by Kaplan-Meier statistics according to the TCGA molecular subgroups (left) and histological subgroups (right).

**Supplementary Figure S2. Analysis of the somatic mutations identified in ECs and SECs, and the characterization of somatic mutations identified by whole exome sequencing (WES).** (**A**) Number of somatic mutations identified in the primary tumor and metastatic regions from the samples. (**B**) Percentage of common somatic mutations identified in the EC sub-groups. (**C**) Percentage of sub-clonal mutations identified by EC sub-groups. Venn diagrams representing the somatic mutations shared among the primary tumor sections and metastatic tissue analyzed by WES. The number of genetic variants are shown and the percentages are in brackets. Phylogenetic trees based on the somatic mutations depicting the evolution of the two primary tumor areas (T) and a metastasis (M). The length of the branches is proportional to the number of shared or private somatic mutations. The pie charts represent the mutational signatures for the shared or private somatic mutations. (**D**) SEC1, (**E**) SEC2 and (**F**) SEC3: *p-value <0.05.

**Supplementary Figure S3.** (**A**) Percentage of somatic mutations shared by all the regions analyzed in each sample by whole exome sequencing (WES, yellow) and targeted sequencing (blue). Validation of the pathogenic somatic mutations identified by targeted sequencing in the primary tumor (T) and metastatic regions (M) of (**B**) SEC3, (**C**) EEC3 and (**D**) EEC4. The mutation types are colored according to the legend and the phylogenetic trees generated according to the presence or absence of the somatic mutations detected in the validation analysis are shown below each case.

**Supplementary Figure S4.** Copy Number Aberrations for serous endometrial carcinomas (SEC) analyzed by Array Comparative Genome Hybridization (aCGH).

**Supplementary Figure S5. (A)** Additional histological and immunohistochemical PDX regions from tumor 2 of the AEC patient implanted in PDX. Magnification 20x**.** (**B**) WES variants identified in the patient derived xenografts (PDXs) obtained from the ambiguous endometrial cancer (AEC) of one patient. Circle chart representing the percentage of variants detected in the WES of the PDX samples. On the right are the variants previously identified in the WES of the AEC patient that were detected in the PDX-WES and on the left, the variants exclusively detected in the PDX-WES study.

**Supplementary Tables:** Table S1, WES sequencing results for all the samples; Table S2, Summary of the total SNVs, insertions and deletions (indels) and non-synonymous SNVs identified in each tumor region analyzed by WES; Table S3, Summary of the samples used for the whole exome sequencing, targeted validation and array comparative genome hybridization (aCGH); Table S4, Validation study, Ampliseq custom panel designs; Table S5, Somatic variants identified by WES in the PDX tumors derived from an ambiguous endometrial carcinoma; Table S6, Selection of the treatment from the in silico study based on information available in the CTD and STITCH databases, and the mutated genes found in the ambiguous endometrial carcinoma; Table S7, Primary antibodies used for the immunohistochemistry analyses.
