# Supplementary material for: Intratumor genetic heterogeneity and clonal evolution to decode endometrial cancer progression
Source: Oncogene. 2022 Feb 10;41(13):1835–50. doi: 10.1038/s41388-022-02221-0 (PMC8956509; doi:10.1038/s41388-022-02221-0)

Supplementary Figure 1

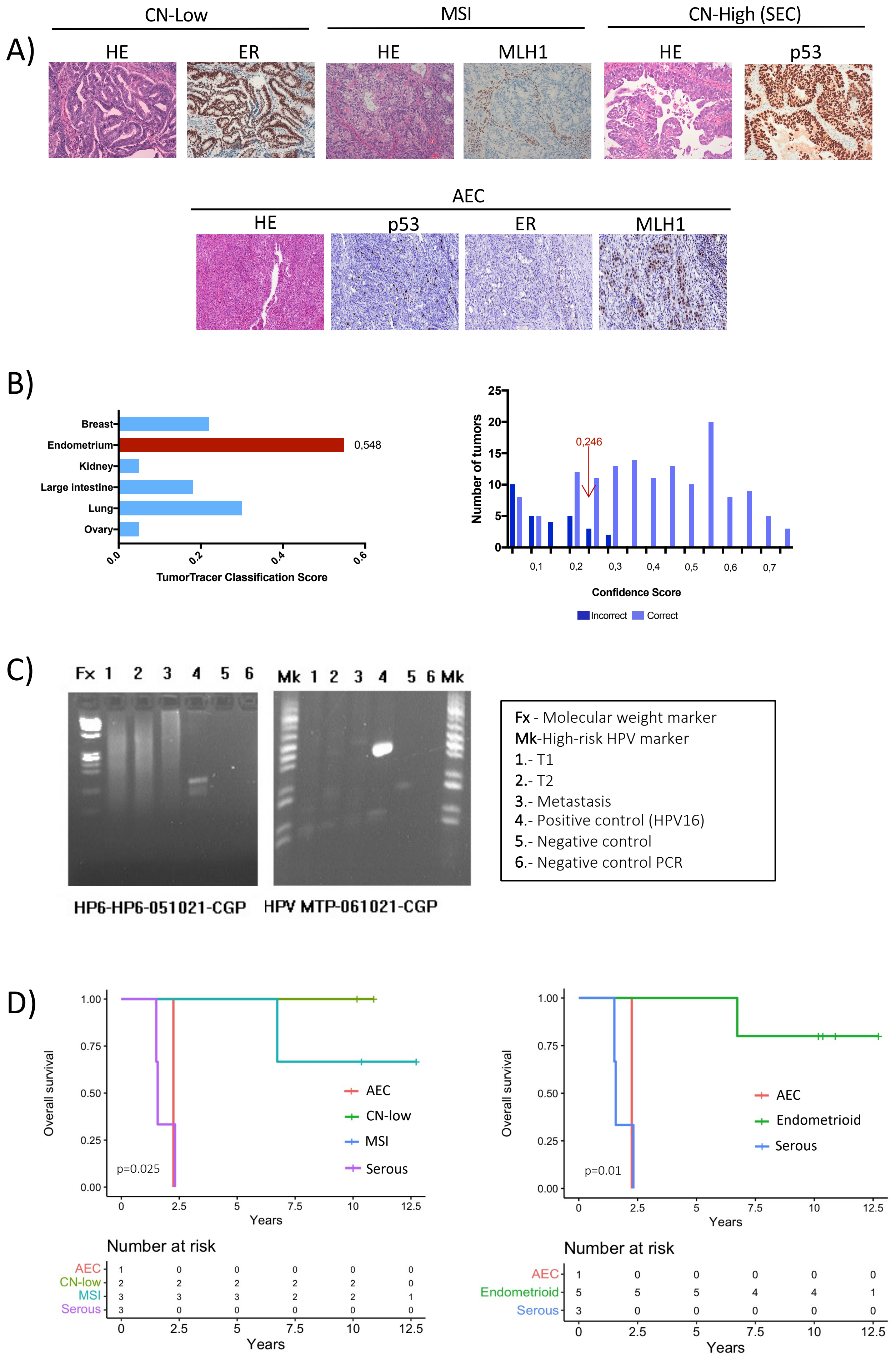

Supplementary Figure 2

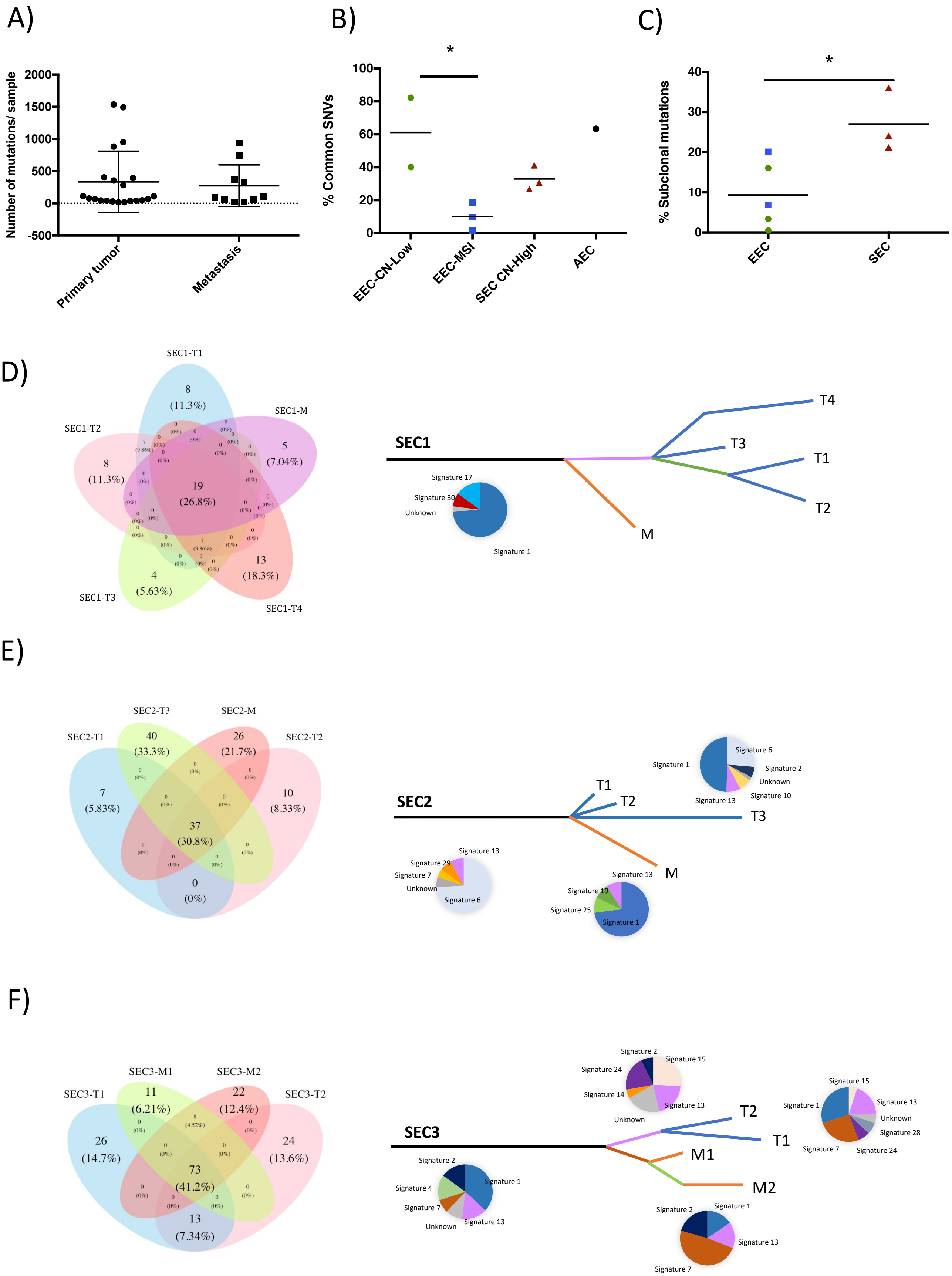

Supplementary Figure 3

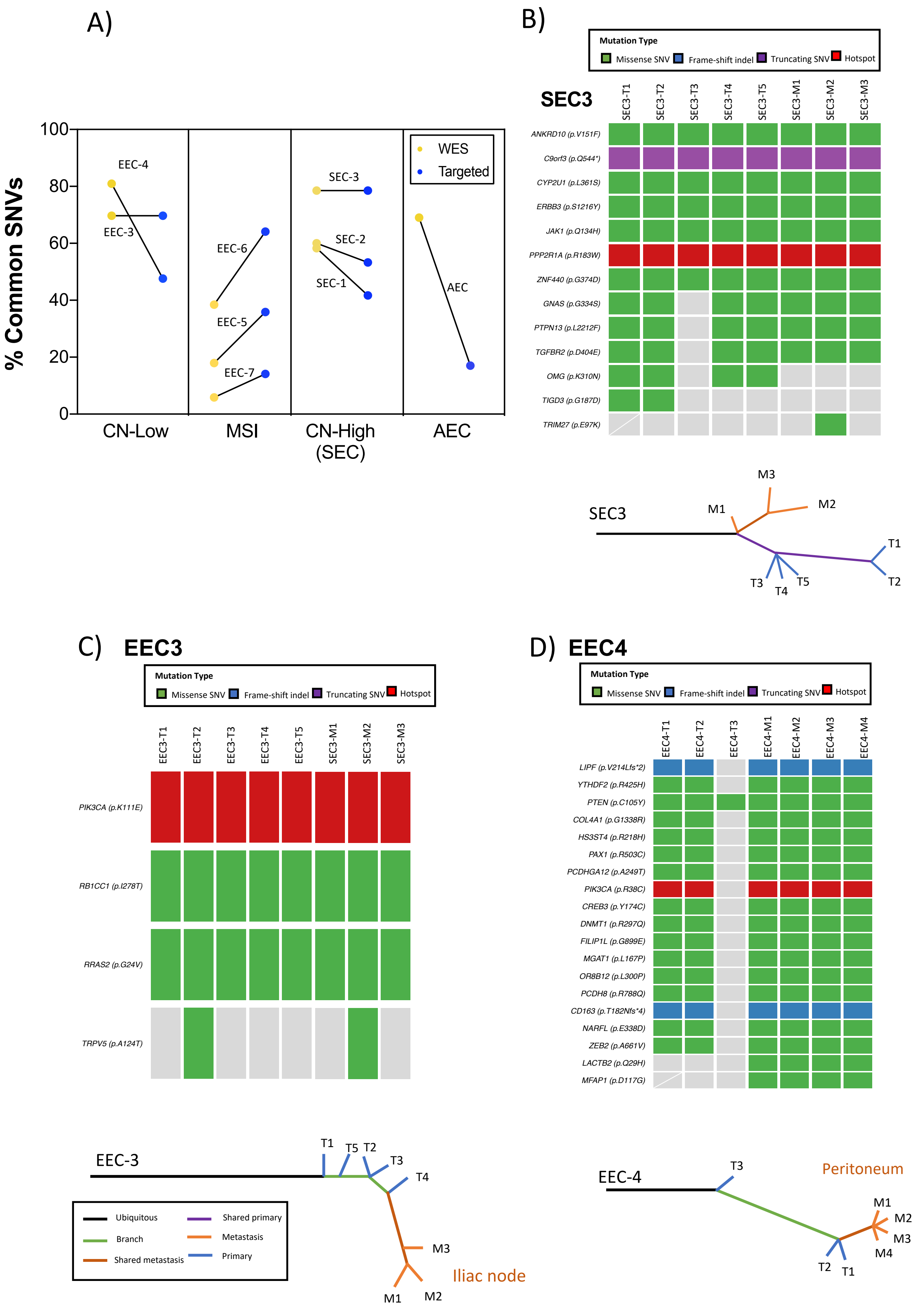

Supplementary Figure 4

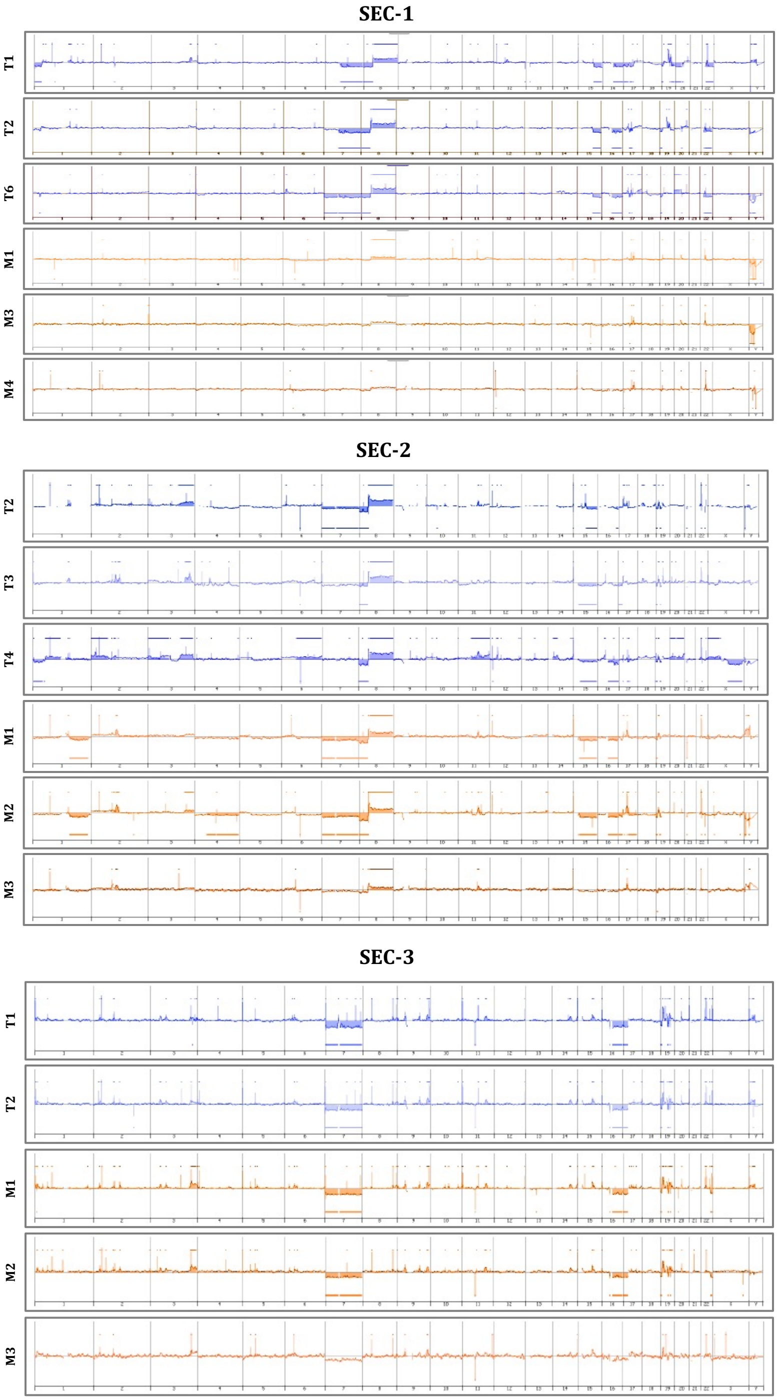

Supplementary Figure 5

A)

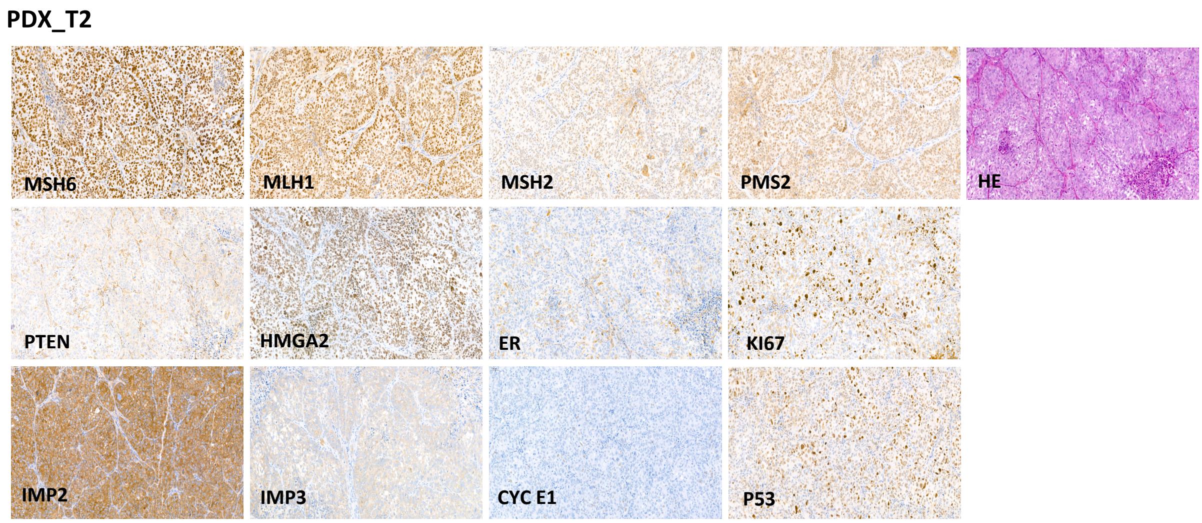

B)

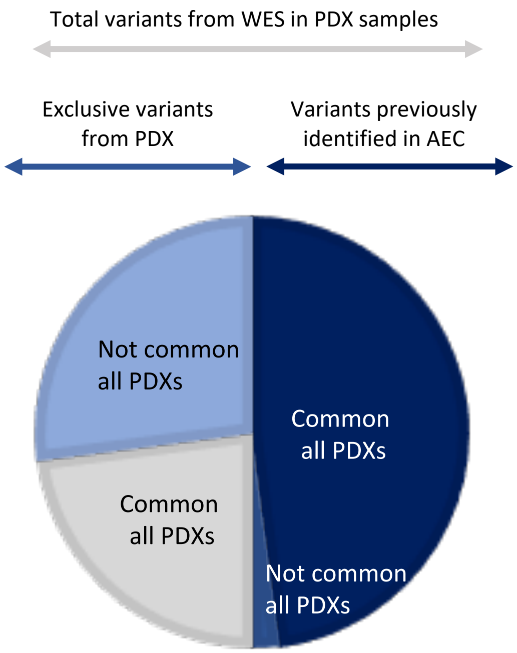

Supplement: Supplementary file 2 — Supplementary Figures 1-6 [file 41388_2022_2221_MOESM2_ESM.pdf]
