# Supplementary material for: Intratumor genetic heterogeneity and clonal evolution to decode endometrial cancer progression
Source: Oncogene. 2022 Feb 10;41(13):1835–50. doi: 10.1038/s41388-022-02221-0 (PMC8956509; doi:10.1038/s41388-022-02221-0)
Supplement: Supplementary file 5 — Supplementary Table S3 [file 41388_2022_2221_MOESM5_ESM.pdf]

Table S3: Summary of the samples used for whole exome sequencing, targeted validation and array comparative genome hybridization (aCGH)

Patient	Sample Name	Type of Tissue	Surgery	Anatomical location	Type of Analysis
EEC-3	EEC-3_T1	Frozen	Primary	Uterus	WES+Validation
EEC-3	EEC-3_T2	Frozen	Primary	Uterus	WES+Validation
EEC-3	EEC-3_N	Frozen	Primary	Uterus	WES+Validation
EEC-3	EEC-3_M	Frozen	Recurrence	Node	WES+Validation
EEC-3	EEC-3_T3	FFPE	Primary	Uterus	Validation
EEC-3	EEC-3_T4	FFPE	Primary	Uterus	Validation
EEC-3	EEC-3_T5	FFPE	Primary	Uterus	Validation
EEC-3	EEC-3_M2	FFPE	Recurrence	Iliac node	Validation
EEC-3	EEC-3_M3	FFPE	Recurrence	Iliac node	Validation
EEC-4	EEC-4_T1	Frozen	Primary	Uterus	WES+Validation
EEC-4	EEC-4_T2	Frozen	Primary	Uterus	WES+Validation
EEC-4	EEC-4_N	Frozen	Primary	Uterus	WES+Validation
EEC-4	EEC-4_M	Frozen	Recurrence	Epiplon	WES+Validation
EEC-4	EEC-4_T3	FFPE	Primary	Uterus	Validation
EEC-4	EEC-4_M2	FFPE	Recurrence	Peritoneum	Validation
EEC-4	EEC-4_M3	FFPE	Recurrence	Peritoneum	Validation
EEC-4	EEC-4_M4	FFPE	Recurrence	Peritoneum	Validation
EEC-5	EEC-5_T1	Frozen	Primary	Depth tumor	WES+Validation
EEC-5	EEC-5_T2	Frozen	Primary	Superficial tumor	WES+Validation
EEC-5	EEC-5_N	Frozen	Primary	Uterus	WES+Validation
EEC-5	EEC-5_M	Frozen	Primary	Peritoneun	WES+Validation
EEC-5	EEC-5_T3	FFPE	Primary	Uterus	Validation
EEC-5	EEC-5_T4	FFPE	Primary	Uterus	Validation
EEC-5	EEC-5_T5	FFPE	Primary	Uterus	Validation
EEC-6	EEC-6_T1	Frozen	Primary	Depth tumor	WES+Validation
EEC-6	EEC-6_T2	Frozen	Primary	Superficial tumor	WES+Validation
EEC-6	EEC-6_N	Frozen	Primary	Uterus	WES+Validation
EEC-6	EEC-6_M	Frozen	Primary	Ovary	WES+Validation
EEC-6	EEC-6_T3	FFPE	Primary	Uterus	Validation
EEC-6	EEC-6_T4	FFPE	Primary	Uterus	Validation
EEC-6	EEC-6_T5	FFPE	Primary	Uterus	Validation
EEC-6	EEC-6_T6	FFPE	Primary	Uterus	Validation
EEC-6	EEC-6_M2	FFPE	Primary	Ovary	Validation
EEC-6	EEC-6_M3	FFPE	Primary	Ovary	Validation
EEC-6	EEC-6_M4	FFPE	Primary	Ovary	Validation
EEC-6	EEC-6_M5	FFPE	Primary	Ovary	Validation
EEC-7	EEC-7_T1	Frozen	Primary	Uterus	WES+Validation
EEC-7	EEC-7_T2	Frozen	Primary	Uterus	WES+Validation
EEC-7	EEC-7_M	Frozen	Recurrence	Diaphragm	WES+Validation
EEC-7	EEC-7_N	Frozen	Recurrence	Blood	WES+Validation
EEC-7	EEC-7_T3	FFPE	Primary	Uterus	Validation
EEC-7	EEC-7_M2	FFPE	Primary	Diaphragm	Validation
EEC-7	EEC-7_M3	FFPE	Primary	Diaphragm	Validation
EEC-7	EEC-7_M4	FFPE	Primary	Diaphragm	Validation
EEC-7	EEC-7_M5	FFPE	Primary	Diaphragm	Validation
SEC-1	SEC-1_T1	Frozen	Primary	Uterus	WES+Validation+aCGH
SEC-1	SEC-1_T2	Frozen	Primary	Uterus	WES+Validation+aCGH
SEC-1	SEC-1_T3	Frozen	Primary	Uterus	WES+Validation
SEC-1	SEC-1_T4	Frozen	Primary	Uterus	WES+Validation
SEC-1	SEC-1_N	Frozen	Primary	Uterus	WES+Validation
SEC-1	SEC-1_M	Frozen	Primary	Ovary	WES+Validation+aCGH
SEC-1	SEC-1_T5	Frozen	Primary	Uterus	Validation
SEC-1	SEC-1_T6	Frozen	Primary	Uterus	Validation+aCGH
SEC-1	SEC-1_T7	Frozen	Primary	Uterus	Validation
SEC-1	SEC-1_M2	FFPE	Primary	Ovary	Validation
SEC-1	SEC-1_M3	FFPE	Primary	Ovary	Validation+aGCH
SEC-1	SEC-1_M4	FFPE	Primary	Tube	Validation+aCGH
SEC-2	SEC-2_T1	Frozen	Primary	Uterus	WES+Validation
SEC-2	SEC-2_T2	Frozen	Primary	Uterus	WES+Validation+aCGH
SEC-2	SEC-2_T3	Frozen	Primary	Uterus	WES+Validation+aCGH
SEC-2	SEC-2_N	Frozen	Primary	Uterus	WES+Validation
SEC-2	SEC-2_M1	Frozen	Primary	Ovary	WES+Validation+aCGH
SEC-2	SEC-2_T4	Frozen	Primary	Uterus	Validation+aCGH
SEC-2	SEC-2_M2	Frozen	Primary	Ovary	Validation+aCGH
SEC-2	SEC-2_M3	FFPE	Primary	Ovary and tube	Validation+aCGH
SEC-2	SEC-2_M4	FFPE	Primary	Ovary	Validation
SEC-3	SEC-3_T1	Frozen	Primary	Uterus	WES+Validation+aCGH
SEC-3	SEC-3_T2	Frozen	Primary	Uterus	WES+Validation+aCGH
SEC-3	SEC-3_M1	Frozen	Primary	Ovary	WES+Validation+aCGH
SEC-3	SEC-3_M2	Frozen	Primary	Ovary	WES+Validation+aCGH
SEC-3	SEC-3_N	Frozen	Primary	Uterus	WES+Validation
SEC-3	SEC-3_T3	FFPE	Primary	Uterus	Validation
SEC-3	SEC-3_T4	FFPE	Primary	Uterus	Validation
SEC-3	SEC-3_T5	FFPE	Primary	Uterus	Validation
SEC-3	SEC-3_M3	FFPE	Primary	Tube	Validation+aGCH
AEC	AEC_T1	Frozen	Primary	Superficial tumor	WES+Validation
AEC	AEC_T2	Frozen	Primary	Superficial tumor	WES+Validation
AEC	AEC_N	Frozen	Primary	Uterus	WES+Validation
AEC	AEC_M	Frozen	Primary	Node	WES+Validation
AEC	AEC_T3	FFPE	Primary	Uterus	Validation
AEC	AEC_T4	FFPE	Primary	Uterus	Validation
AEC	AEC_T5	FFPE	Primary	Uterus	Validation

N: normal, T: primary tumor, M: metastasis, WES: whole exome sequencing, Torrent System Validation: targeted massive parallel sequencing using ion Torrent System
