# Supplementary material for: Intratumor genetic heterogeneity and clonal evolution to decode endometrial cancer progression
Source: Oncogene. 2022 Feb 10;41(13):1835–50. doi: 10.1038/s41388-022-02221-0 (PMC8956509; doi:10.1038/s41388-022-02221-0)
Supplement: Supplementary file 8 — Supplementary Table S6 [file 41388_2022_2221_MOESM8_ESM.docx]

| **MUTATED GENES** | **RELATED DRUGS** | **DATABASE** |
| --- | --- | --- |
| *CYP1A2, TICRR, MAP2K4, GSPT1, ATM, DYNC1I1* | Bortezomib | CTD |
| *CYP1A2, MAP2K4* | Bortezomib | STITCH |
| *CYP1A2, MAP2K4, NAV3, TOP2B* | Paclitaxel | CTD |
| *CYP1A2, TICRR, MAP2K4, GSPT1, ATM, DYNC1I1, NAV3, TOP2B* | Bortezomib  Paclitaxel | CTD/STITCH |
| *ATM* | Nucleic Acid Synthesis Inhibitors  Bortezomib | CTD |

**Table S6.** Treatment selection from the *in silico* study based on information available in the CTD and STITCH databases, and the genes mutated in the ambiguous endometrial carcinoma (AEC).
